# 4′‐ThioRNA Substitution and Sense‐Strand Segmentation for the Design of Functional Fully Modified siRNAs Targeting SARS‐CoV‐2

**DOI:** 10.1002/chem.71507

**Published:** 2026-08-03

**Authors:** Yuhei Nogi, Jun Tsukimoto, Noriko Saito‐Tarashima, Takaaki Koma, Masako Nomaguchi, Noriaki Minakawa

**Affiliations:** ^1^ Graduate School of Pharmaceutical Science Tokushima University Tokushima Tokushima Japan; ^2^ Department of Microbiology, Graduate School of Medicine Tokushima University Tokushima Tokushima Japan; ^3^ Division of Interdisciplinary Research for Medicine and Photonics, Institute of Post‐LED Photonics Tokushima University Tokushima Tokushima Japan

**Keywords:** 4'‐thioRNA, chemical modifications, modification pattern, SARS‐CoV‐2, siRNA

## Abstract

Chemically modified small interfering RNAs (siRNAs) are proving to be highly effective as therapeutic agents, but the extent and positioning of chemical modifications must be carefully tuned because the RNA interference (RNAi) machinery imposes intrinsic constraints on siRNA architecture. Here, using a SARS‐CoV‐2‐targeting siRNA as a model, we have systematically investigated the positional and extent‐dependent tolerance of 4′‐thioRNA substitution within functional siRNAs. Single 4′‐thioRNA substitution was broadly tolerated across multiple positions, and complete substitution of either the sense or antisense strand preserved robust RNAi activity. In contrast, full substitution of both strands resulted in a pronounced loss of activity. Physicochemical analysis suggested that this loss arises from interference with cleavage and removal of the sense strand during loading of the RNA‐induced silencing complex (RISC). Sense‐strand segmentation was therefore applied to fully 4′‐thio‐modified siRNA to mimic the post‐cleavage state during RISC loading. This design partially restored RNAi activity, facilitating the development of fully 4'‐thio‐modified siRNAs with antiviral activity against SARS‐CoV‐2. Collectively, these findings expand the chemical space available for functional siRNA design.

## Graphical abstract

4′‐ThioRNA was broadly tolerated across multiple positions in SARS‐CoV‐2‐targeting siRNAs, but full 4'‐thio modification of both strands caused a reduction in RNAi activity. Sense‐strand segmentation improved RNAi and antiviral activity, particularly when the nick was located at position 10 from the 5′‐end of the sense strand, thereby unlocking the function of fully 4′‐thio‐modified siRNAs.

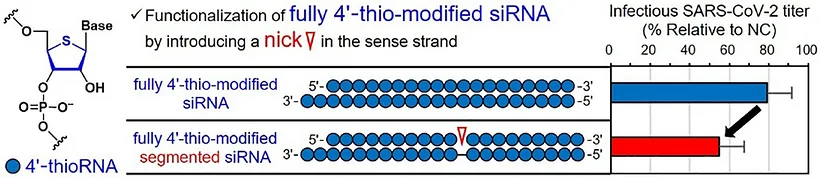

## 1 Introduction

Small interfering RNAs (siRNAs) have matured into a clinically validated therapeutic modality, with many approved drugs demonstrating the power of RNA interference (RNAi) for sequence‐specific gene silencing [1, 2]. A central requirement for therapeutic siRNAs is extensive chemical modification to enhance nuclease resistance, increase affinity for target mRNA, and improve pharmacokinetic properties [3]. Clinically successful siRNAs incorporate combinations of multiple nucleotide modifications, most notably 2′‐*O*‐methyl (2′‐OMe) and 2′‐deoxy‐2′‐fluoro (2′‐F) ribonucleotides, as well as phosphorothioate linkages, often implemented within the framework known as “enhanced stabilization chemistry (ESC)” [4, 5]. While siRNAs modified by these chemistries are proving to be highly effective [4, 5, 6], the RNAi machinery imposes intrinsic constraints, necessitating careful optimization of the extent and positioning of each modification. For example, heavy incorporation of 2′‐F nucleotides has been associated with increased potential toxicity [7], motivating partial substitution with 2′‐OMe nucleotides. However, excessive or improperly positioned 2′‐OMe modification may introduce steric interference with substrate recognition by Argonaute (Ago), a core component of the RNA‐induced silencing complex (RISC), thereby disrupting RISC loading and/or strand selection and ultimately diminishing RNAi potency [5, 8, 9]. Similarly, extensive incorporation of phosphorothioate linkages is thought to adversely affect silencing activity and increase toxicity [10, 11, 12, 13], further underscoring the importance of optimizing both the type and placement of chemical modifications.

4′‐ThioRNA is a ribose‐modified RNA analogue in which the ring oxygen at the 4′‐position is replaced by sulfur (Figure 1a) [14, 15]. Originally developed to enhance nuclease resistance while preserving RNA‐like structural features, 4′‐thioRNA has since been explored as a versatile platform for nucleic acid therapeutics. We have previously expanded the synthetic strategies of 4′‐thioRNA and demonstrated that 4′‐thioRNA confers excellent nuclease resistance while preserving RNA‐like structural features [14]. We also showed its compatibility with diverse oligonucleotide modalities, including siRNAs [16, 17], RNA aptamers [14, 18], anti‐microRNA oligonucleotides [19, 20], and guide RNA for the CRISPR–Cas9 system [21]. Those studies have consistently suggested that 4′‐thioRNA exhibits an unusual propensity for substitution tolerance without severely perturbing native RNA function. In the context of siRNAs, however, previous studies have examined only limited extents of 4′‐thioRNA substitution [16, 17]; and the extent to which 4′‐thioRNA substitution may be accommodated within functional siRNAs remains unresolved. Addressing this issue is relevant not only for the design of chemically stabilized siRNAs but also for gaining insight into the tolerance of the RNAi machinery to systematic backbone and sugar modifications.

**FIGURE 1:**
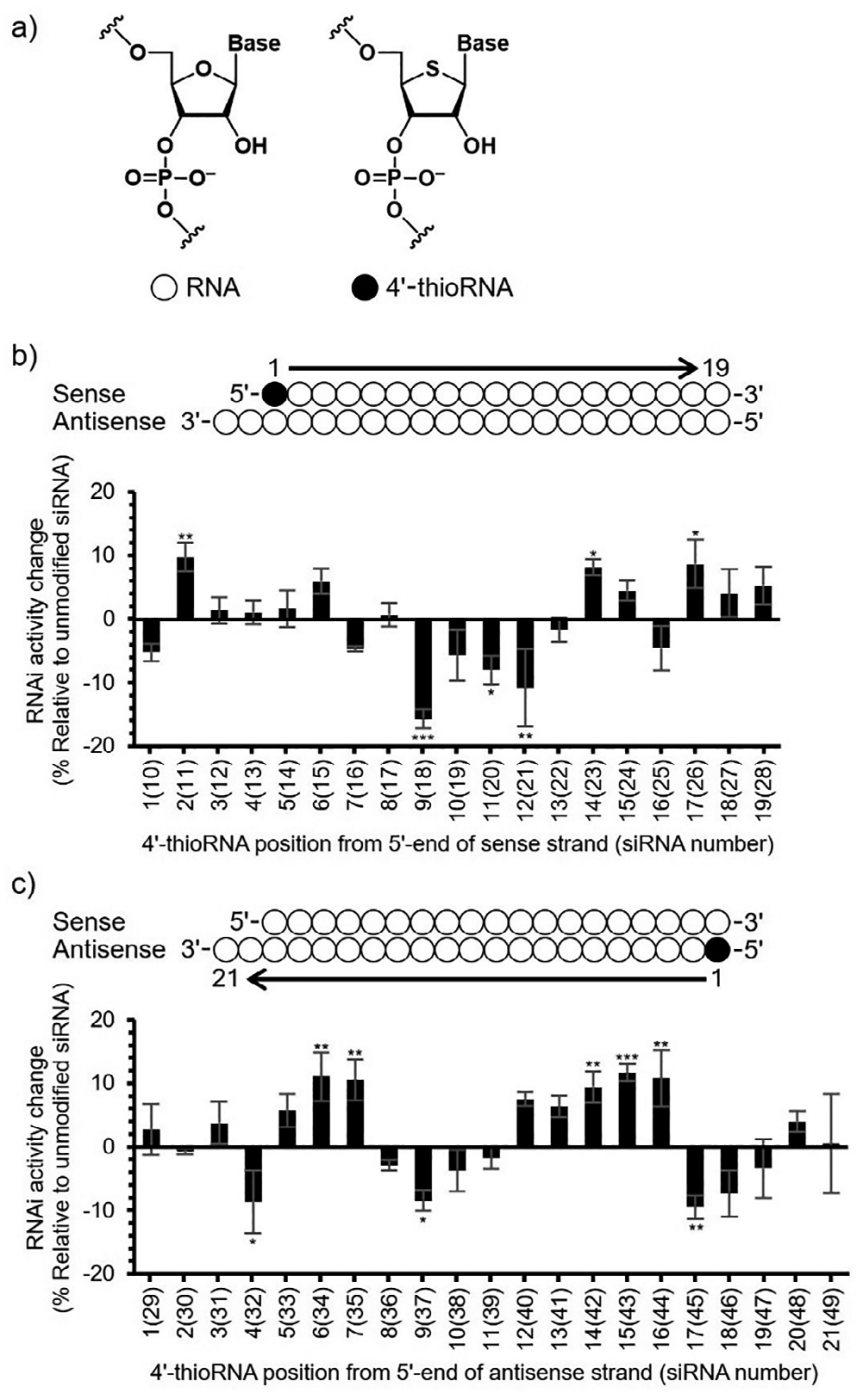
Structure of 4′‐thioRNA and positional effects of single 4′‐thioRNA substitutions in a SARS‐CoV‐2 RdRp‐targeting siRNA. (a) Chemical structures of RNA and 4′‐thioRNA. (b,c) Changes in RNAi activity of siRNAs containing a single 4′‐thioRNA substitution at the indicated position in the sense (b) or antisense (c) strand of the SARS‐CoV‐2 RdRp‐targeting parental siRNA, relative to the unmodified siRNA (**si5**). RNAi activity was evaluated at 9.00 nM using a luciferase reporter assay in Vero E6 cells stably expressing a reporter transcript bearing the target sequence in the 3′‐UTR. Positive and negative values indicate, respectively, increased and decreased RNAi activity as compared with the unmodified siRNA. Data are shown as mean ± SD from three independent replicates (*n* = 3). Statistical significance versus unmodified **si5** was assessed by one‐way ANOVA followed by Dunnett's multiple comparisons test: ^*^ *p* < 0.05, ^**^ *p* < 0.01, ^***^ *p* < 0.001.

To explore this subject, in this study, we employed an siRNA targeting the RNA‐dependent RNA polymerase (RdRp) of SARS‐CoV‐2 as a model system and systematically introduced extensive 4′‐thioRNA substitutions. During viral infection, the intracellular abundance of viral RNA increases dramatically, creating a highly competitive environment for RNAi‐mediated silencing; this system therefore provided a stringent benchmark for evaluating the functional compatibility of extensively modified siRNAs. We performed RNAi reporter assays, antiviral assays, thermal melting (*T*_m_) analysis, and circular dichroism (CD) spectroscopy to define the functional and physicochemical consequences of 4′‐thioRNA incorporation into siRNAs. We then applied a sense‐strand segmentation strategy to address the constraints associated with extensive modification and to evaluate 4′‐thioRNA as a platform for chemically stabilized siRNA design.

## 2 Results

### 2.1 Selection of a SARS‐CoV‐2‐Targeting Model siRNA

The positive‐sense RNA genome of SARS‐CoV‐2 contains numerous potential target sites for siRNAs [22], which may be broadly classified into regions unique to the genomic RNA (gRNA) and regions shared between the gRNA and subgenomic RNAs (sgRNAs) generated during discontinuous transcription [23]. Previous studies have shown that siRNAs directed to gRNA‐specific regions generally exhibit stronger antiviral activity than those targeting regions common to both gRNA and sgRNAs [24], probably because the high abundance of sgRNAs produced during viral replication [25] may sequester siRNAs and reduce effective target engagement.

Taking these observations into consideration, we surveyed siRNAs targeting several gRNA‐specific regions of the SARS‐CoV‐2 genome, drawing on sequences reported in the literature [24, 26, 27]. Various candidate siRNAs (**si1**–**si9**) were evaluated in cytopathic effect (CPE) assays in SARS‐CoV‐2‐infected Vero E6 cells (Figure S1). Among the candidates, **si5** targeting RNA‐dependent RNA polymerase (RdRp; Nsp12) consistently exhibited robust antiviral activity. RdRp is an essential and highly conserved component of the viral replication–transcription machinery [28] and has been extensively validated as a functional antiviral target, for example, by the clinically used nucleoside analogues remdesivir [29, 30] and molnupiravir [31, 32]. Therefore, **si5** was selected for subsequent chemical modification studies.

### 2.2 Synthesis of 4′‐Thio‐modified siRNAs

The components of 4′‐thio‐modified siRNAs (**si10**–**si62, si66**–**si68**, and **sisi75**–**sisi78**) were synthesized using standard solid‐phase phosphoramidite chemistry with previously reported 4′‐thioRNA building blocks [14]. After deprotection and purification, the identity of each 4′‐thio‐modified oligonucleotide was confirmed by LC–MS analysis (Table S1).

### 2.3 Positional and Extent‐Dependent Tolerance of 4′‐ThioRNA Substitution in siRNAs

To define the positional tolerance of 4′‐thioRNA substitution in siRNAs, we first examined the effects of single substitutions. We prepared a series of siRNAs in which a single nucleotide of the parental **si5** was replaced with a 4′‐thioRNA residue, either in the sense strand (**si10**–**si28**) or in the antisense strand (**si29**–**si49**) (Figure 1b,c and Table S2). RNAi activity was measured by using a luciferase assay in Vero E6 cells stably expressing a reporter transcript harboring the siRNA target sequence in the 3′‐UTR. Activity changes were calculated relative to the unmodified **si5** duplex, with positive and negative values indicating increased and decreased activity, respectively. At 9.00 nM, single 4′‐thioRNA substitutions in the sense strand (**si10**–**si28**) were generally well tolerated; however, substitutions at positions 9–12 from the 5′‐end of the sense strand (**si18**–**si21**) caused a modest reduction in RNAi activity. Single substitutions in the antisense strand (**si29**–**si49**) were likewise well tolerated overall, with only slight decreases observed at positions 4 (**si32**), 9 (**si37**), and 17 (**si45**) from the 5′‐end. Notably, substitutions at positions 6, 7, and 14–16 from the 5′‐end of the antisense strand (**si34**, **si35**, and **si42–si44**) enhanced RNAi activity. Thus, single 4′‐thioRNA substitutions seem to be broadly tolerated across the siRNA duplex.

Next, we examined the effects of multiple 4′‐thioRNA substitutions. siRNAs bearing clustered substitutions within defined regions of either the sense or antisense strand (**si50**–**si55**) were prepared, and their half‐maximal inhibitory concentration (IC_50_) values were determined (Figure 2). All multiply 4′‐thio‐modified siRNAs tested had IC_50_ values of 7.26–9.71 nM, comparable to that of the unmodified **si5** duplex (8.55 nM), indicating that 4′‐thioRNA substitution remains well tolerated even when approximately one‐third of either strand is consecutively replaced within a localized region. Notably, **si51**, which carried a cluster of 4′‐thioRNA substitutions in the middle of the sense strand, retained potency comparable to that of unmodified **si5** (IC_50_ = 8.63 vs. 8.55 nM), despite the modest effects observed for single substitutions at positions 9–12 in this region (**si18**–**si21**, Figure 1b). We further evaluated alternating modification patterns in which approximately half of the nucleotides in either the sense or antisense strand were replaced with 4′‐thioRNA residues (**si56**–**si59**, Figure 2). These siRNAs showed only modest reductions in RNAi potency, with IC_50_ values (10.8–16.4 nM) still within an order of magnitude of the unmodified **si5** (8.55 nM). Thus, extensive but periodic 4′‐thioRNA substitution remains compatible with efficient RNAi.

**FIGURE 2:**
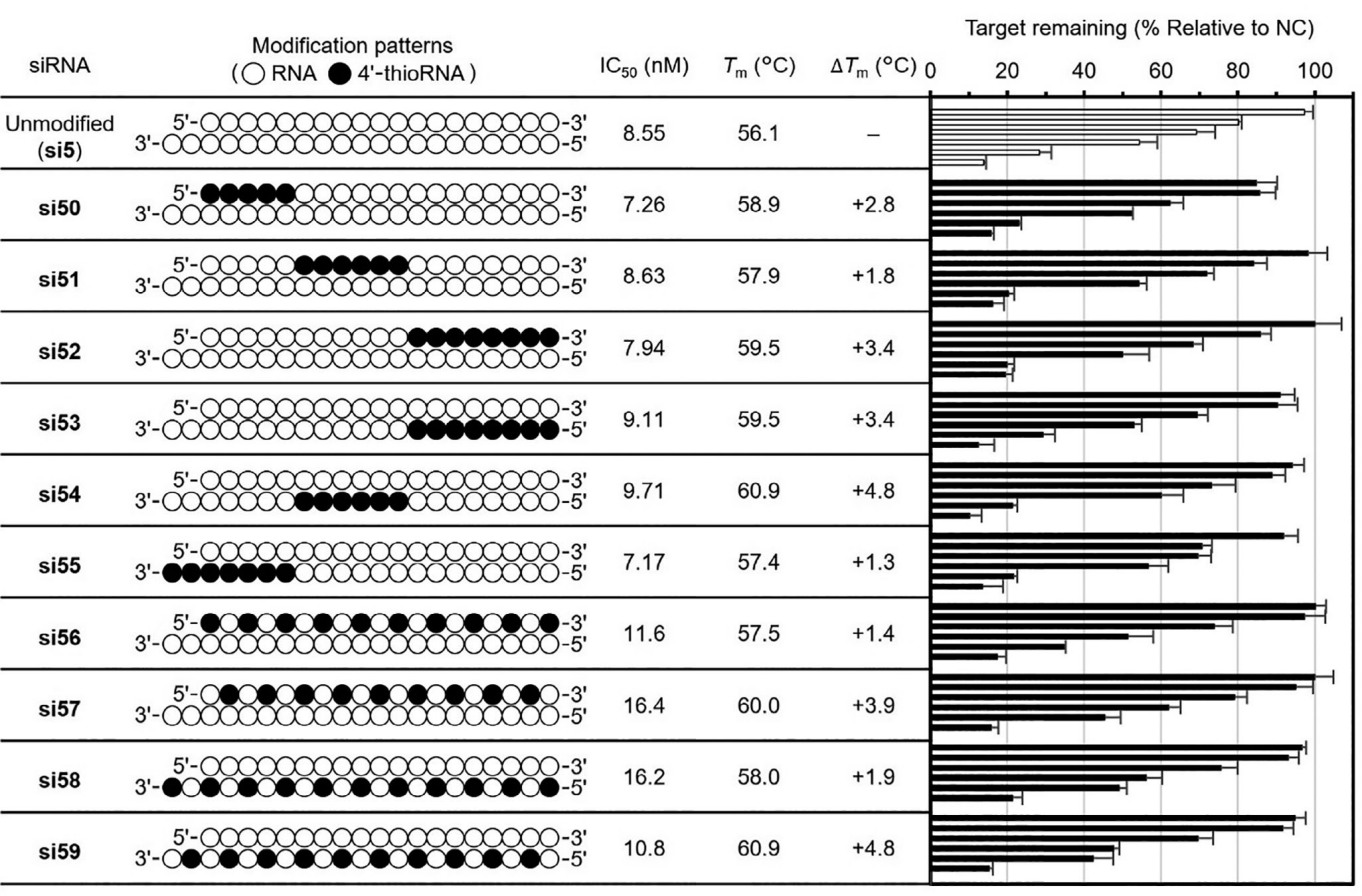
RNAi activity and thermal stability of siRNAs with approximately one‐third clustered or alternating 4′‐thioRNA substitutions. Both clustered and alternating 4′‐thioRNA substitution patterns were generally well tolerated, with RNAi activity comparable to that of unmodified **si5**, even when approximately one‐third to one‐half of either strand was substituted. IC_50_ values were determined with siRNA concentrations ranging from 0.100 to 50.0 nM. Bar graphs represent RNAi activity at siRNA concentrations of 0.100, 1.00, 3.00, 9.00, 30.0, and 50.0 nM, arranged from top to bottom. Data are shown as mean + SD from three independent replicates (*n* = 3). *T*_m_ values were determined for siRNA duplexes (1.00 µM) in 0.1 × PBS. ∆*T*_m_ values were calculated relative to unmodified **si5**.

We also examined the effect of complete strand substitution with 4′‐thioRNA (Figure 3). siRNAs in which either the sense or antisense strand was fully replaced with 4′‐thioRNA (**si60** and **si61**) retained substantial RNAi activity with IC_50_ values (9.79 and 12.4 nM, respectively) close to that of unmodified **si5** (8.55 nM). For comparison, siRNAs possessing complete 2′‐OMe substitution in either strand were also evaluated. Full 2′‐OMe substitution of the sense strand (**si63**) preserved partial activity (IC_50_ = 23.1 nM), whereas full substitution of the antisense strand (**si64**) almost completely abolished activity (IC_50_ > 50.0 nM). In contrast, the activity of an siRNA fully substituted with 4′‐thioRNA in both strands (**si62**) was markedly reduced (IC_50_ > 50.0 nM).

**FIGURE 3:**
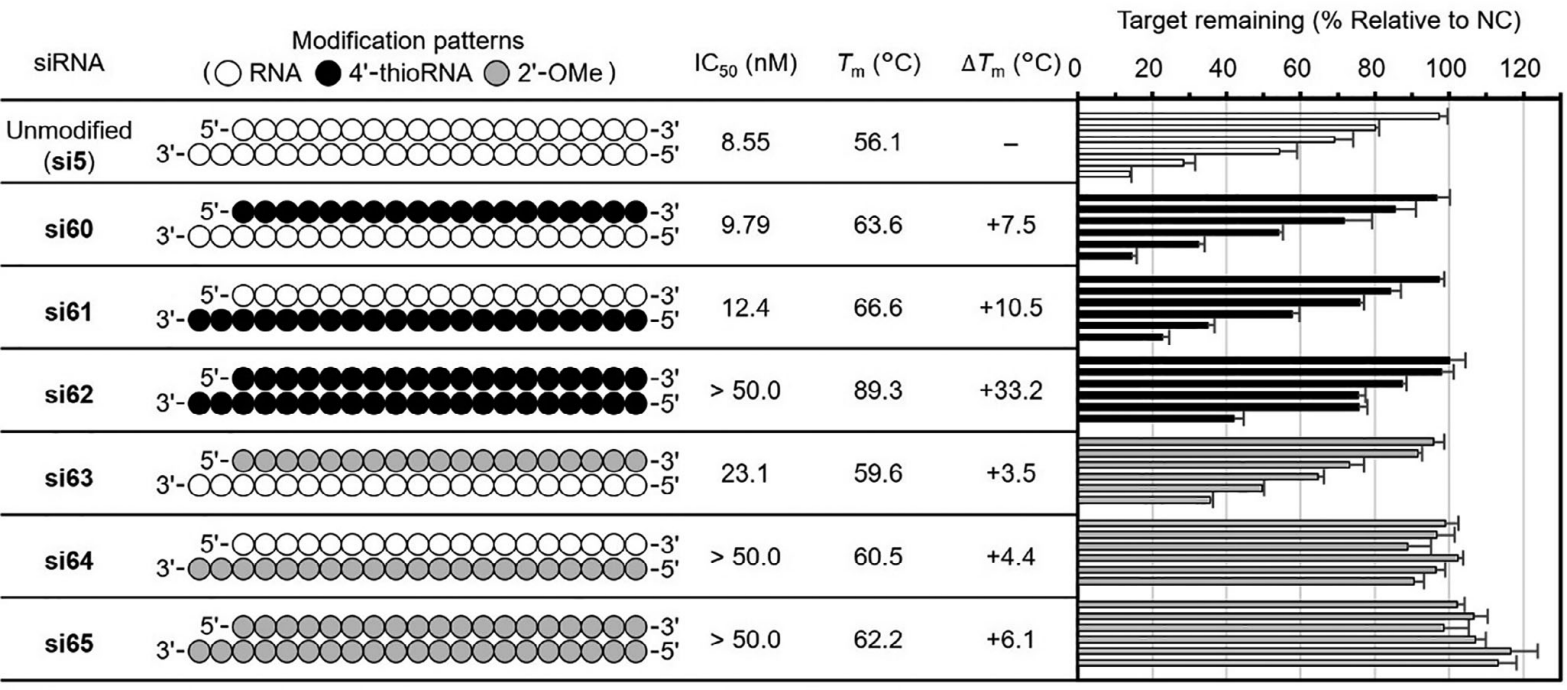
RNAi activity and thermal stability of siRNAs that were fully substituted with 4′‐thioRNA or 2′‐OMe in one or both strands. Complete substitution with 4′‐thioRNA in either the sense or antisense strand (**si60** and **si61**) was well tolerated, but there was a marked loss of activity when both strands were substituted (**si62**); full 2′‐OMe substitution in the antisense strand (**si64**) severely impaired RNAi activity. IC_50_ values were determined with siRNA concentrations ranging from 0.100 to 50.0 nM. Bar graphs represent RNAi activity at siRNA concentrations of 0.100, 1.00, 3.00, 9.00, 30.0, and 50.0 nM, arranged from top to bottom. Data are shown as mean + SD from three independent replicates (*n* = 3). *T*_m_ values were determined for siRNA duplexes (1.00 µM) in 0.1 × PBS. ∆*T*_m_ values were calculated relative to unmodified **si5**.

### 2.4 Physicochemical Characterization of 4′‐Thio‐modified siRNA Duplexes

Because 4′‐thioRNA is known to form stable duplexes with both natural RNA and itself [14], we examined its effect on duplex stability in the present siRNA system by measuring *T*_m_. Single 4′‐thioRNA substitutions (**si10**–**si49**) caused only slight stabilization, with Δ*T*_m_ values of +0.0 to +2.8°C relative to unmodified **si5** (Table S2). More extensive substitution (**si50**–**si61**) produced larger increases in *T*_m_, with Δ*T*_m_ values of +1.3 to +10.5°C (Figures 2 and 3). A similar but more moderate trend was observed for fully 2′‐OMe‐modified siRNAs (**si63**–**si65**; Δ*T*_m_ = +3.5 to +6.1°C). Among all duplexes examined, **si62**, in which both strands were fully substituted with 4′‐thioRNA, showed a marked increase in duplex stability (Δ*T*_m_ = +33.2°C).

To test whether this unusually high thermal stability contributes to the reduced RNAi activity of **si62**, we introduced a destabilizing (*S*)‐glycol nucleic acid ([*S*]‐GNA) residue into the antisense strand [33, 34]. For siRNAs **si66**–**si68**, each containing a single (*S*)‐GNA residue at position 6, 7, or 8 from the 5′‐end of the antisense strand in the fully 4′‐thio‐modified background, *T*_m_ values were reduced by 3.4°C–10.5°C relative to the parental **si62** duplex (Figure 4a). However, this destabilization did not restore activity; instead, **si66**–**si68** were less active than **si62**. In contrast, the same (*S*)‐GNA substitutions were well tolerated in unmodified siRNA (**si69**–**si71**; Figure S2) and caused only slight activity loss when introduced into siRNAs bearing a 4′‐thio‐modified antisense strand (**si72**–**si74**).

**FIGURE 4:**
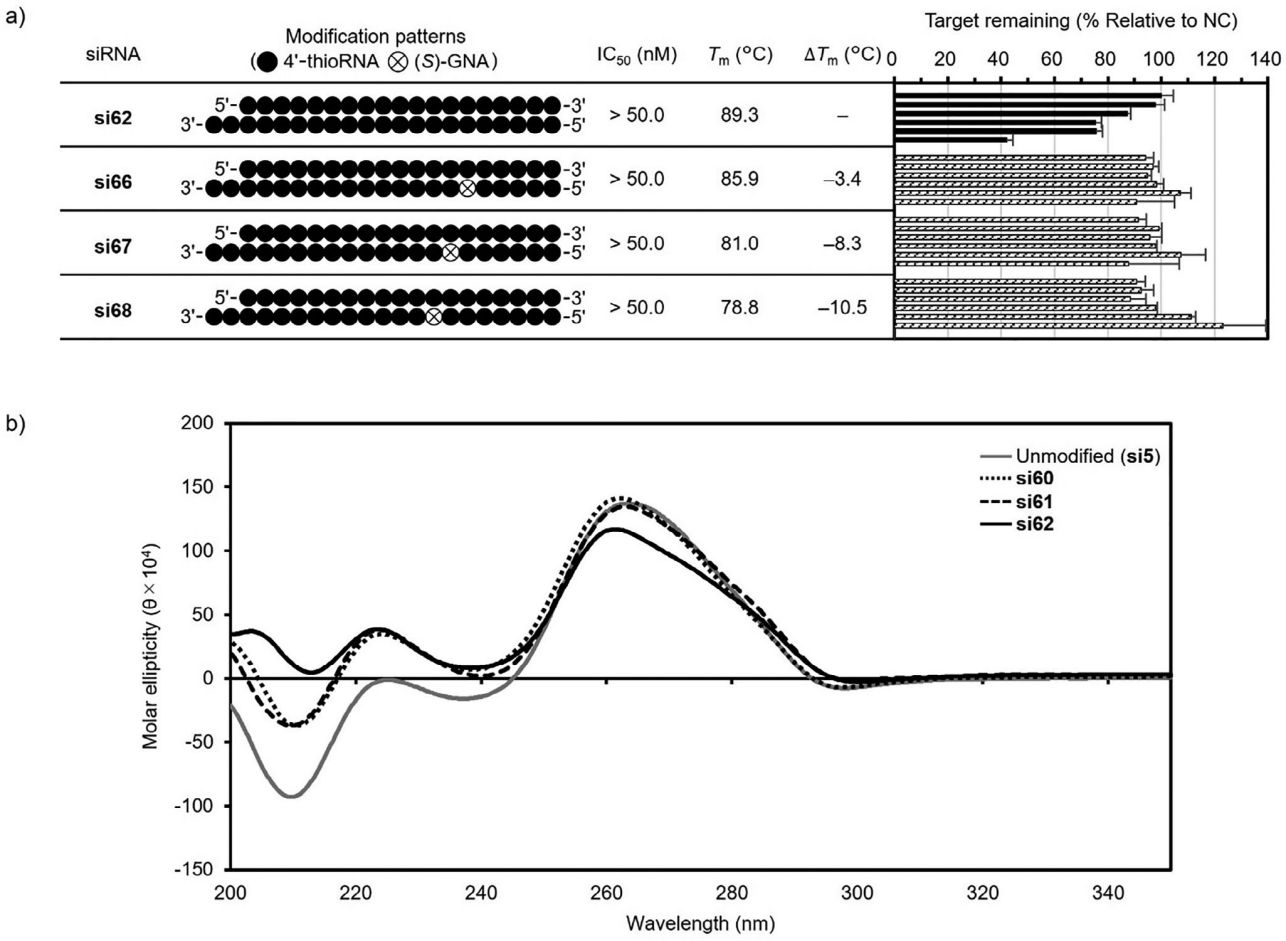
Evaluation of RNAi activity upon reduction of thermal stability by a single (*S*)‐GNA substitution in **si62**, and structural analysis of siRNA duplexes with 4′‐thioRNA substitutions. (a) RNAi activity and thermal stability of fully 4′‐thio‐modified siRNAs containing a single (*S*)‐GNA substitution at position 6, 7, or 8 from the 5′‐end of the antisense strand. Introduction of a (*S*)‐GNA residue into **si62** reduced thermal stability but did not restore RNAi activity, indicating that the excessive duplex stability of **si62** is not the sole cause of activity loss. IC_50_ values were determined with siRNA concentrations ranging from 0.100 to 50.0 nM. Bar graphs represent RNAi activity at siRNA concentrations of 0.100, 1.00, 3.00, 9.00, 30.0 and 50.0 nM, arranged from top to bottom. Data are shown as mean + SD from three independent replicates (*n* = 3). *T*_m_ values were determined for siRNA duplexes (1.00 µM) in 0.1 × PBS. ∆*T*_m_ values were calculated relative to **si62**. (b) CD spectra of unmodified **si5** and siRNA duplexes 4′‐thio‐modified on one or both strands (**si60**–**si62**). All duplexes retained A‐form‐like features, while the fully 4′‐thio‐modified **si62** exhibited a measurable change in the overall spectral profile with a clear reduction in molar ellipticity around 210 nm. Spectra were recorded for siRNA duplexes (2.00 µM) in 1×PBS.

Since the fully 4′‐thio‐modified duplexes **si60**–**si62** showed distinct activity profiles, we evaluated their global conformations by CD spectroscopy. The CD spectra of unmodified **si5** and the single‐strand 4′‐thio‐modified duplexes **si60** and **si61** were characteristic of an A‐form duplex, with a positive band around 260 nm and a negative band around 210 nm (Figure 4b). **si62**, in which both strands were fully substituted with 4′‐thioRNA, also retained an A‐form‐like spectrum, but its molar ellipticity was reduced, particularly near 210 nm. In addition, the spectrum of **si62** crossed those of **si5**, **si60**, and **si61** near 280 nm, suggesting that **si62** adopts distinct overall conformational features.

### 2.5 Sense‐Strand Segmentation of Fully 4′‐Thio‐modified siRNA Restores RNAi Activity

To further investigate the reasons for reduced activity of the fully 4′‐thio‐modified **si62** duplex, we prepared small internally segmented interfering RNAs (sisiRNAs) containing a nick in the middle of the sense strand [35]. Fully 4′‐thio‐modified sisiRNAs (**sisi75**–**sisi78**) were constructed by introducing a nick at position 8, 9, 10, or 11 from the 5′‐end of the sense strand. The segmented duplexes retained CD spectra similar to that of **si62**, indicating that the nick did not substantially alter the global duplex conformation (Figure S3). In contrast, all segmented duplexes exhibited lower *T*_m_ values than **si62** (Figure S4). In RNAi assays, the fully 4′‐thio‐modified sisiRNAs (**sisi75**–**sisi78**) were more active than **si62** (IC_50_ = 19.8–34.4 vs. > 50.0 nM) (Figure 5). Among them, **sisi76** and **sisi77**, bearing nicks at positions 9 and 10 from the 5′‐end, showed the strongest recovery of activity, with IC_50_ values (21.7 and 19.8 nM, respectively) approaching that of unmodified **si5** (8.55 nM).

**FIGURE 5:**
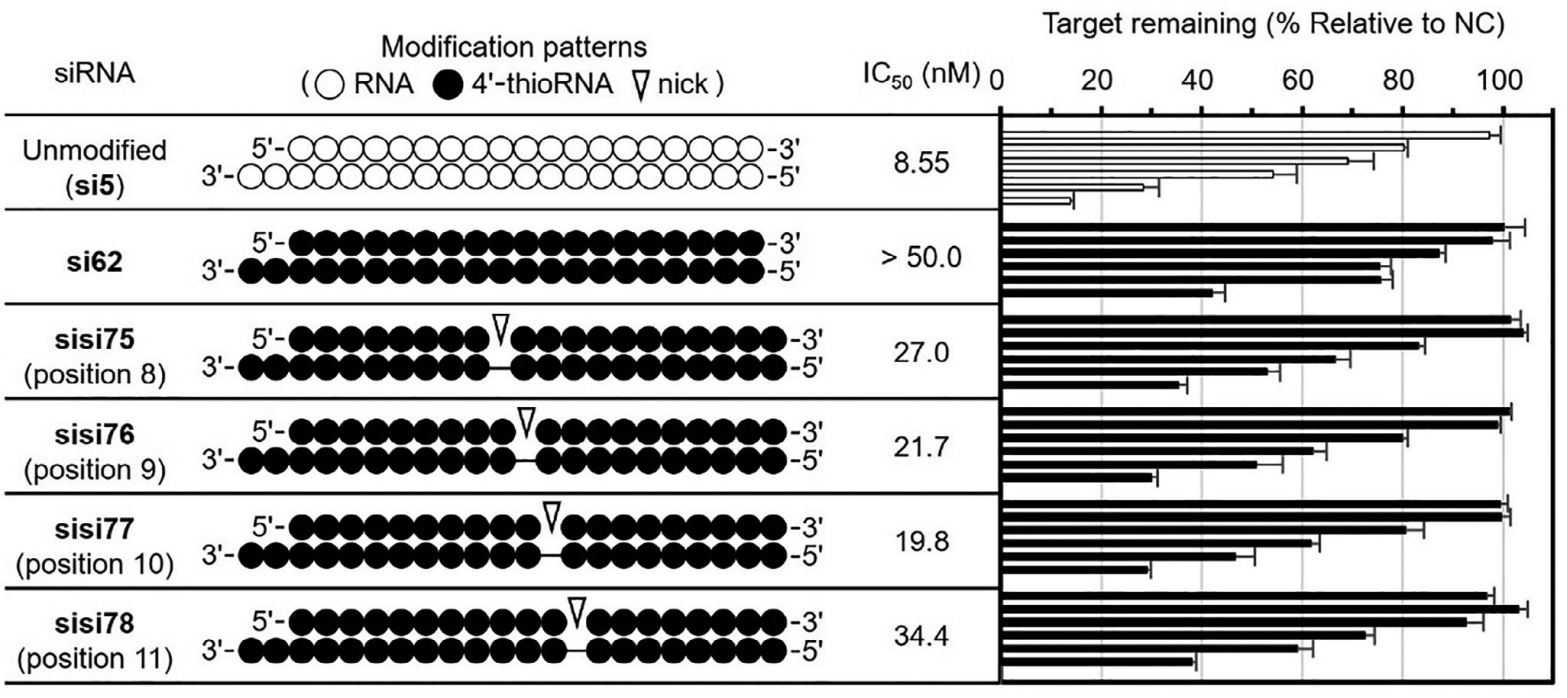
RNAi activity of fully 4′‐thio‐modified sisiRNAs containing a nick at position 8, 9, 10, or 11 from the 5′‐end of the sense strand. Introduction of a nick into the sense strand restored RNAi activity in a position‐dependent manner, with maximal recovery observed at positions 9 and 10 (**sisi76** and **sisi77**), corresponding to the canonical Ago2 cleavage site. IC_50_ values were determined with siRNA concentrations ranging from 0.100 to 50.0 nM. Bar graphs represent RNAi activity at siRNA concentrations of 0.100, 1.00, 3.00, 9.00, 30.0, and 50.0 nM, arranged from top to bottom. Data are shown as mean + SD from three independent replicates (*n* = 3).

Next, to assess whether this improvement reflected reduced sense‐strand‐derived off‐target effects [35], we performed reporter assays (Figure S5). As sisiRNA architecture was originally developed by Bramsen et al. [35] to suppress sense‐strand loading, we tested whether differential sense‐strand incorporation into RISC contributed to the observed effects. However, neither **si62** nor the sisiRNA constructs showed detectable silencing activity arising from the sense strand, indicating that the restored activity of segmented **sisi75**–**sisi78** is unlikely to result from reduced sense‐strand off‐target effects.

Given the previously established ability of 4′‐thioRNA to enhance nuclease resistance in oligonucleotides [14], we next examined whether this property is retained in 4′‐thio‐modified siRNA duplexes and, in particular, whether it is compromised by introduction of a central nick in the sense strand. To this end, Cy5‐labeled unmodified **si5** and 4′‐thio‐modified siRNAs (**si60**–**si62**, **sisi76**, and **sisi77**) were incubated in 80% FBS at 37°C, and the remaining intact siRNA was analyzed by 15% native polyacrylamide gel electrophoresis (Figure S6). Compared with unmodified **si5**, the 4′‐thio‐modified **si60**–**si62** exhibited increased resistance to serum nuclease degradation in a modification‐dependent manner. Among the segmented fully 4′‐thio‐modified constructs, **sisi76** and **sisi77**, which showed the most effective RNAi activity, exhibited minimal degradation even after prolonged incubation in serum. These results indicate that sense‐strand segmentation provides access to fully 4′‐thio‐modified siRNAs that combine substantial RNAi activity with high serum stability.

### 2.6 Antiviral Activity Against SARS‐CoV‐2

Lastly, we evaluated the antiviral activity of the modified siRNAs by plaque assay to assess infectious virus production and reverse transcription‐quantitative polymerase chain reaction (RT‐qPCR) using a primer set targeting SARS‐CoV‐2 RdRp RNA. Vero E6 cells were transfected with siRNA (30.0 nM) for 24 h and then infected with SARS‐CoV‐2 at a multiplicity of infection (MOI) of 0.01. Culture supernatants were collected two days after infection and subjected to plaque assays using fresh Vero E6 cells, as well as to RT‐qPCR analysis. The unmodified **si5** duplex reduced plaque formation by approximately 70% (Figure 6), while **si60**, in which the sense strand was fully substituted with 4′‐thioRNA, showed a comparable antiviral effect. By contrast, **si61**, containing a fully 4′‐thio‐modified antisense strand, reduced plaque formation by only 17%. Likewise, fully modified **si62** reduced plaque formation by about 21%, confirming a marked loss of antiviral activity. Introduction of a central nick into the sense strand (**sisi75**–**sisi78**) partially improved antiviral potency. **sisi77**, bearing a nick at position 10 from the 5′‐end, exhibited the strongest antiviral activity among the segmented constructs, reducing plaque formation by 45%, consistent with the trend observed in our reporter assay results. RT‐qPCR analysis further supported the antiviral effects observed in the plaque assay (Figure S7).

**FIGURE 6:**
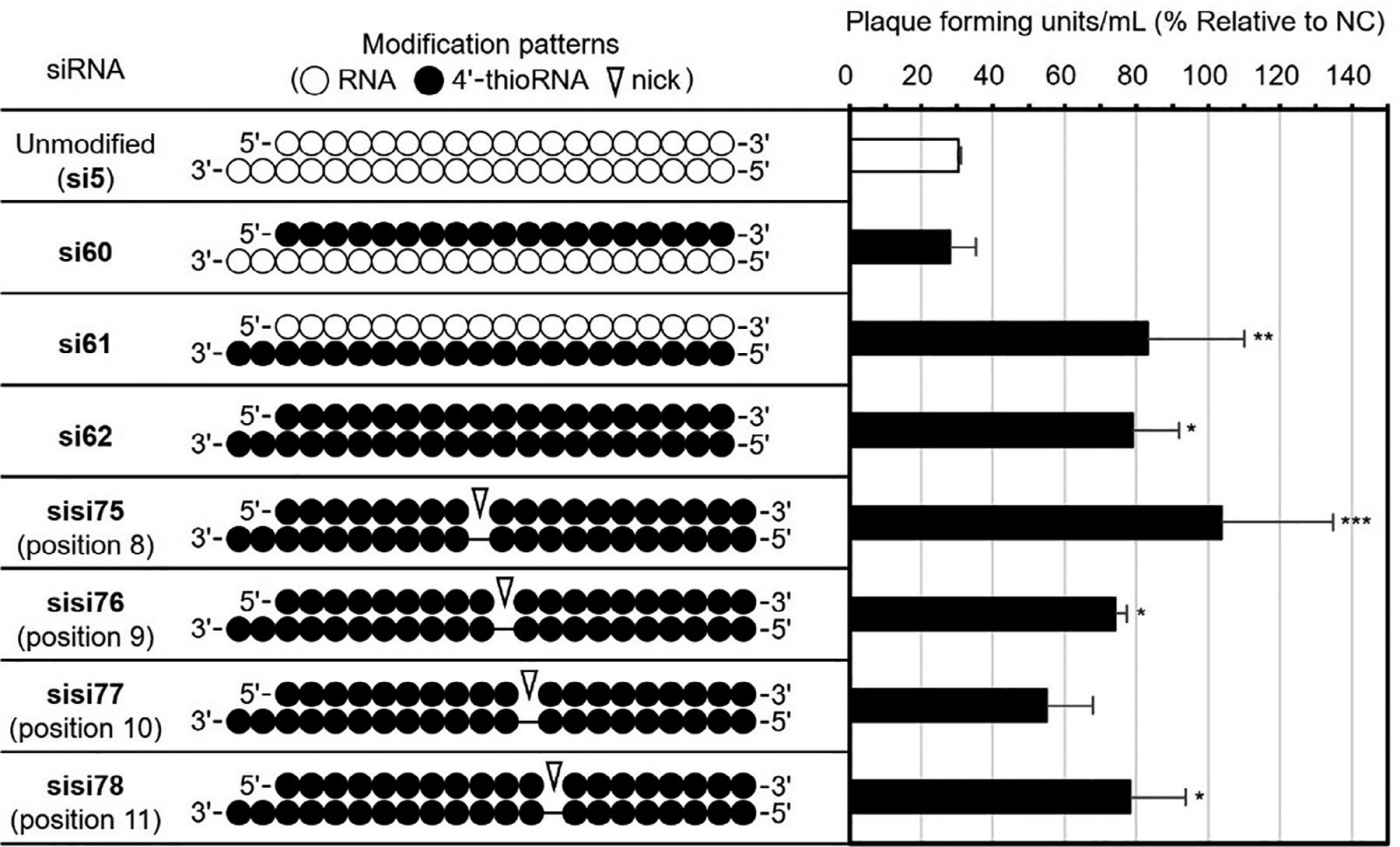
Anti‐SARS‐CoV‐2 activity of 4′‐thio‐modified siRNAs and sisiRNAs. Sense‐strand substitution (**si60**) was well tolerated, whereas antisense or full duplex substitution (**si61** and **si62**) markedly reduced antiviral activity. Activity was partially restored by introduction of a central nick in a position‐dependent manner, with the strongest antiviral effect observed at position 10 from the 5′‐end of the sense strand (**sisi77**), consistent with the reporter assay. Vero E6 cells were pre‐transfected with 30.0 nM siRNA for 24 h and then infected with SARS‐CoV‐2 at an MOI of 0.01. Supernatants were collected 2 days after infection and used to determine viral titers by plaque assay on fresh Vero E6 cells. At 3 dpi, the cells were fixed and stained with crystal violet, and the number of plaques was counted to calculate plaque‐forming units. Data are shown as mean + SD from three independent replicates (*n* = 3). Statistical significance versus unmodified **si5** was assessed using one‐way ANOVA followed by Dunnett's multiple comparisons test: ^*^ *p* < 0.05, ^**^ *p* < 0.01, ^***^ *p* < 0.001.

## 3 Discussion

This study has revealed the positional and extent‐dependent tolerance of 4′‐thio‐modifications in a functional antiviral siRNA targeting the SARS‐CoV‐2 RdRp (Nsp12) region. An RdRp target site located within a gRNA‐specific region was selected because siRNAs directed to sequences unique to gRNA have been shown to outperform those targeting regions shared with abundant sgRNAs during infection [24]. Using the RdRp targeting **si5** as the parental unmodified duplex, we systematically evaluated the tolerance of 4′‐thio modifications across the duplex in a luciferase reporter assay and found that RNAi activity was retained over a broad range of substitution patterns. Single substitutions with 4′‐thioRNA (**si10**–**si49**) were tolerated at nearly all positions (Figure 1b,c), and even periodic or clustered substitutions (**si50**–**si59**) maintained strong silencing activity with only modest changes in IC_50_ (Figure 2). Complete replacement of either the sense or antisense strand with 4′‐thioRNA (**si60** and **si61**) also preserved robust activity. This level of strand‐wide tolerance differs from the behavior commonly observed for many 2′‐modifications such as 2′‐OMe, which may be poorly tolerated at some positions and leads to substantially reduced activity when extensively introduced into the antisense strand [5, 8, 9]. Collectively, our observations suggest that 4′‐thioRNA expands the chemical space compatible with functional siRNA design.

A pronounced loss of activity emerged only when both strands were fully substituted with 4′‐thioRNA. Duplexes containing full substitution in only one strand (**si60** and **si61**) retained efficient silencing, whereas substitution across both strands (**si62**) resulted in a pronounced loss of activity (Figure 3). This transition suggests that the determining factor is not simply the total number of modified residues, but a property that becomes dominant only after full duplex conversion. One possible explanation is excessive duplex stabilization, because 4′‐thioRNA is known to increase affinity for the complementary strand [14] and progressively raised duplex *T*_m_ in our measurements. Indeed, the fully substituted duplex **si62** showed the highest *T*_m_ among the constructs examined (Figure 3). However, reducing duplex stability by introducing a (*S*)‐GNA residue into the antisense strand (**si66**–**si68**) did not improve activity and instead further reduced potency despite lowering *T*_m_ (Figure 4a). These observations indicate that increased thermal stability alone does not account for the loss of activity, and imply that the fully substituted duplex acquires an additional property that limits productive RNAi.

Our previous structural studies have shown that nucleic acid duplexes modified at the 4′‐position can exhibit characteristic structural features distinct from those of natural duplexes. For example, a fully 4′‐thio‐modified DNA duplex was shown by NMR analysis to adopt an RNA‐like A‐form helix [36]. In addition, X‐ray crystallographic analysis of a related 4′‐seleno‐modified RNA duplex revealed that it retains an overall A‐form helical architecture, while the modified 4′‐seleno sugar can adopt an unusual *south*‐type conformation [37]. Based on these precedents, we examined by CD spectroscopy whether complete replacement of all nucleotides in the siRNA duplex with 4′‐thioRNA affects the overall conformational features of the duplex (Figure 4b).

The fully substituted duplex **si62** exhibited a distinct CD spectral profile compared with the unmodified duplex **si5** and the single‐strand fully modified duplexes **si60** and **si61**, including a clear reduction in molar ellipticity, particularly around 210 nm. At the same time, the overall spectral features remained broadly consistent with an A‐form‐like duplex. Since CD spectroscopy does not define local structural parameters such as sugar conformation, hydrogen bonding, or base‐stacking geometry, these spectral changes should be interpreted cautiously. Thus, the altered CD spectrum of **si62** suggests that full 4′‐thioRNA substitution of both strands is associated with distinct conformational features at the duplex level, without specifying a particular local structural change. The lack of similar spectral changes in **si60** and **si61**, in which only one strand was fully substituted, further suggests that this duplex‐level conformational feature becomes apparent only upon complete replacement of both strands.

We next considered which step of the RNAi pathway might be sensitive to these duplex‐level conformational features. Productive loading of RISC onto the antisense strand requires removal of the sense strand; and in the canonical pathway, Ago2 cleaves the sense strand between nucleotides 9 and 10 from its 5′ end [38, 39]. To test whether bypassing this step might restore silencing, we used a sisiRNA architecture, originally developed by Bramsen et al. [35] to reduce sense‐strand‐derived off‐target effects, in which the sense strand is segmented. This design restored activity of the fully 4′‐thioRNA substituted duplex, and the extent of recovery depended on the position of the nick (Figure 5). A nick introduced at position 9 (**sisi76**) or 10 (**sisi77**) produced the strongest rescue, consistent with their proximity to the canonical Ago2 cleavage site and the geometry expected after sense‐strand cleavage. The CD spectra of the fully 4′‐thio‐modified sisiRNA duplexes (**sisi75**–**sisi78**) remained essentially indistinguishable from that of the fully substituted parental duplex (**si62**), indicating that restoration of activity does not require reversal of the global conformational change detected by CD analysis (Figure S3). In addition, assays designed to detect sense‐strand delivered off‐target activity did not reveal detectable silencing from the sense strand in either the parental 4′‐thio‐modified duplex (**si62**) or the sisiRNA constructs (**sisi75**–**sisi78**) (Figure S4). Collectively, these results indicate that the improvement in activity is unlikely to arise from reduced strand‐loading competition, and instead support a model in which full duplex substitution interferes with an early cleavage‐dependent step during RISC loading, most plausibly sense‐strand processing.

Serum stability assays showed that the enhanced nuclease resistance conferred by 4′‐thio‐modification was retained even after introduction of a central sense‐strand nick (Figure S6). In particular, **sisi76** and **sisi77**, which exhibited efficient of RNAi activity, showed minimal degradation in 80% FBS. Thus, sense‐strand segmentation provides access to fully 4′‐thio‐modified siRNAs that combine restored RNAi activity with high serum stability.

Antiviral plaque assays and RT‐qPCR analysis of RdRp RNA levels provided further insight into how these mechanistic constraints affect siRNA function under conditions of infection (Figures 6 and S7). The unmodified RdRp‐targeting **si5** reduced plaque formation by approximately 70%, and the **si60** duplex containing a fully 4′‐thioRNA substituted sense strand produced a similar reduction, consistent with the strong tolerance of sense‐strand modification observed in our reporter assays. In contrast, **si61** with a fully 4′‐thioRNA substituted antisense strand showed only limited plaque reduction despite displaying near‐parental potency in the luciferase reporter assay system. These results indicate that reporter‐based RNAi potency does not necessarily translate directly to antiviral performance for all chemotypes and may reflect infection‐specific factors. Differences in intracellular RNA abundance [25], localization of viral replication, compartmentalization of replication complexes [40, 41], and infection‐associated changes in RNAi pathway dynamics may all contribute to RNAi potency. In addition, the abundance and turnover of viral RNAs, including sgRNAs, may influence effective siRNA engagement as compared with a stable reporter transcript in an in vitro assay even when the target sequence is identical.

The fully 4′‐thioRNA substituted duplex (**si62**), which was strongly impaired in the reporter assay, also showed weak plaque suppression, indicating that the functional boundary associated with full duplex substitution is maintained across experimental contexts. Introducing a nick into the sense strand of the fully substituted duplex (**sisi75**–**sisi78**) improved antiviral efficacy and reproduced the positional trend observed in the gene‐silencing experiments, with nicks closer to the canonical cleavage site providing greater benefit. This agreement supports our interpretation that inefficient sense‐strand processing during RISC loading may limit activity in both contexts, and that structural bypass strategies may partially restore function under infection conditions. Although the activity of the rescued 4′‐thio‐modified sisiRNA did not completely reach that of unmodified **si5** under the conditions tested, the improvement demonstrates that architectural modification of the duplex may substantially enhance the performance of otherwise poorly active fully modified siRNAs.

Our results identify 4′‐thioRNA as a modification with unusually broad compatibility with siRNA function, maintaining RNAi activity under extensive substitution and even full modification of a single strand. At the same time, the sharp reduction in activity observed after full duplex substitution with 4′‐thioRNA reveals a discrete functional boundary that probably arises from cooperative structural effects influencing early RISC‐loading events. Restoration of activity by sense‐strand segmentation provides a practical design strategy to overcome this limitation and suggests that combining 4′‐thioRNA chemistry with control of duplex architecture may facilitate the design of highly modified siRNAs that retain biological activity. Future studies examining Ago2‐mediated sense‐strand processing, strand loading efficiency, and the structural properties of fully substituted duplexes will help to clarify the molecular basis of this boundary and guide the design of antiviral and therapeutic siRNAs.

## 4 Conclusion

In this study, we systematically evaluated the extent to which 4′‐thioRNA substitution can be accommodated within functional siRNAs. Single 4′‐thioRNA substitutions were broadly tolerated across the siRNA duplex without substantially impairing RNAi activity (**si10**–**si49**). Multiple substitutions were also well tolerated (**si50**–**si59**), and duplexes with full substitution of either the sense (**si60**) or antisense strand (**si61**) retained RNAi potency comparable to that of the unmodified siRNA. In contrast, siRNA in which both strands were fully substituted (**si62**) exhibited a marked reduction in activity, indicating the presence of a functional boundary for complete duplex modification. Although full 4′‐thioRNA substitution increased duplex thermal stability (**si62**), reduction of *T*_m_ by introduction of a single (*S*)‐GNA residue did not restore RNAi activity (**si66**–**si68**), suggesting that increased thermal stability alone may not fully account for the loss of activity. Together with the CD analysis, these results are consistent with the possibility that full 4′‐thioRNA substitution alters conformational features of the siRNA duplex as a whole. Sense‐strand segmentation based on the sisiRNA design substantially restored RNAi activity, with the extent of restoration depending on the position of the nick (**sisi75**–**sisi78**). These findings suggest that the fully 4′‐thio‐modified siRNA duplex may be less effectively processed within the RNAi machinery, potentially at a step related to sense‐strand cleavage, and that introduction of a nick may bypass this limitation. Importantly, fully 4′‐thio‐modified sisiRNA, **sisi77**, exhibited efficient anti‐SARS‐CoV‐2 activity, demonstrating that extensive 4′‐thioRNA substitution is compatible with functional antiviral siRNA design. Taken together, our findings demonstrate that 4′‐thio modification can be incorporated into siRNAs to a far greater extent than previously recognized, thereby expanding the chemical space available for functional siRNA design. They further suggest that other 4′‐thio‐modified nucleic acid analogs may represent promising candidates for the future development of chemically stabilized siRNAs.

## 5 Experimental Section

### 5.1 Chemically Modified Oligonucleotide Synthesis

All chemically modified oligonucleotides were synthesized on an automated T‐6 DNA/RNA synthesizer (Nihon Techno Service, Ushiku, Ibaraki, Japan) using the corresponding phosphoramidite units and CPG resin at a 0.4 µmol scale, according to a previously reported standard procedure [42]. Natural RNA and 2′‐OMe phosphoramidites were purchased commercially. 4′‐thioRNA [14] and (*S*)‐GNA [43] phosphoramidites were synthesized according to previously reported protocols. After synthesis, the resulting oligonucleotides were cleaved from the solid support using AMA (1:1 [*v/v*] aqueous ammonium hydroxide and aqueous methylamine) or methanolic ammonia at room temperature, followed by deprotection under AMA conditions at 65°C for 15 min. For Cy5‐labeled oligonucleotides, cleavage from the solid support and deprotection were performed using 28% aqueous ammonia at room temperature for 12 h. The RNA oligonucleotides were dried in a centrifugal evaporator, and the TBDMS groups protecting the 2′‐hydroxyl groups were removed with triethylamine trihydrofluoride at 65°C for 2.5 h. The crude oligonucleotides were purified by a C18 cartridge column, and additionally by reverse‐phase HPLC if required. The resulting oligonucleotides were characterized by LC‐MS analysis. Natural siRNAs (**si1**–**si9**) were purchased from FASMAC (Kanagawa, Japan).

### 5.2 In Vitro Screening Using Vero E6 Cells Stably Expressing Luciferase

The HSV‐TK promoter and *firefly* luciferase coding sequence were PCR‐amplified from the psiCHECK2 vector (Promega) and cloned into the BglII and NotI restriction sites of pcDNA3.1 Hygro(–) (Thermo Fisher Scientific). Subsequently, the siRNA target sequence (5′‐AACTGTCAAACCCGGTAATTT‐3′) was inserted into the NotI and BamHI restriction sites located in the 3′‐UTR of the *firefly* luciferase gene. The resulting reporter vector was transfected into Vero E6 cells (RRID:CVCL_0574, CRL‐1586; ATCC, VA, USA), and stable clones expressing *firefly* luciferase were selected using hygromycin B. The cells were cultured at 37°C and 5% CO_2_ in MEM (Nacalai Tesque, Kyoto, Japan) supplemented with 10% fetal bovine serum (FBS). Cells were seeded in a 96‐well plate at a density of 1 × 10^4^ cells per well. After 24 h, they were transfected with 0.01–5 pmol of siRNA using 0.003–1.5 µL of Lipofectamine RNAiMAX (Thermo Fisher Scientific), made up to 10 µL per well with Opti‐MEM, according to the manufacturer's protocol. RNAi activity was evaluated by measuring *firefly* luciferase activity 48 h after transfection using a luciferase assay system.

### 5.3 Thermal Melting Experiments

Ultraviolet absorbance was measured in 1 cm pathlength quartz cells using a UV‐1800 spectrophotometer equipped with a temperature controller (SHIMAZU, Kyoto, Japan). Melting curves of siRNA duplexes (1.00 µM) were monitored at 260 nm in 0.1 × PBS (13.7 mM NaCl, 0.27 mM KCl, 1.0 mM Na_2_HPO_4_, 0.18 mM KH_2_PO_4_, pH 7.4). Before measurement, samples were heated to 95°C and then slowly cooled to 20°C. Thermal denaturation was performed by heating from 20°C to 95°C at a rate of 0.5°C min^−1^. Melting temperatures (*T*_m_) were calculated from the first derivatives of the heating curves; values are the average of three experiments.

### 5.4 CD Spectroscopy

CD spectra were obtained on a J1500 spectropolarimeter (Jasco, Tokyo, Japan). Samples containing siRNA duplexes (2.00 µM) in 1 × PBS (137 mM NaCl, 2.7 mM KCl, 10 mM Na_2_HPO_4_, 1.8 mM KH_2_PO_4_, pH 7.4) were prepared. Spectra were recorded from 350 to 200 nm at 25°C with a bandwidth of 1 nm, a sampling wavelength of 0.2 nm, and a scanning rate of 50 nm/min. Each spectrum was an average of five scans. The molar ellipticity was calculated from the equation [*q*] = *q/cl*, where *q* is ellipticity (mdeg), *c* is sample concentration, and *l* is cell path length (cm).

### 5.5 Measurement of Sense‐Strand Off‐Target Effects in the Luciferase Reporter Assay

Sense strand off‐target reporter plasmids were constructed by inserting the sequence complementary to the siRNA sense strand (5′‐AAATTACCGGGTTTGACAG‐3′) into the psiCHECK2 vector (Promega) between the Xho1 and Not1 restriction sites. Vero E6 cells were seeded in a 96‐well plate at a density of 1 × 10^4^ cells per well. After 24 h, they were transfected with 0.9 pmol of siRNA and 100 ng of reporter plasmid using 0.27 µL of Lipofectamine RNAiMAX (Thermo Fisher Scientific) for siRNA and 0.3 µL of Lipofectamine 2000 (Thermo Fisher Scientific) for the reporter plasmid, each made up to 10 µL per well with Opti‐MEM, according to the manufacturer's protocols. At 48 h post‐transfection, *firefly* luciferase activity and *Renilla* luciferase activity were measured using a dual‐luciferase assay system.

### 5.6 Nuclease Resistance Experiments

Cy5‐labeled siRNAs (10.0 pmol) were incubated at 37°C in a solution containing 80% FBS and 1 × PBS in a total volume of 100 µL. Before incubation, the siRNAs were heated to 95°C and then slowly cooled to room temperature. At the indicated time points (0, 15, and 30 min and 1, 3, 6, 12, and 24 h), 10 µL aliquots were collected and mixed with 5 µL of 0.5 M EDTA to quench the reaction. Glycerol (5 µL) was then added, and the samples were analyzed by electrophoresis on a 15% native polyacrylamide gel. The gels were visualized using a Typhoon FLA 9500 scanner (Cytiva, Tokyo, Japan), and band intensities were quantified using ImageQuant TL software (Cytiva). The percentage of intact siRNA remaining at each time point was determined from the band intensities relative to the 0 min sample.

### 5.7 Antiviral Assays

Vero E6 cells were seeded in a 96‐well plate at a density of 3 × 10^4^ cells per well. After 24 h, they were transfected with 3 pmol of siRNA using 0.9 µL of Lipofectamine RNAiMAX (Thermo Fisher Scientific), made up to 10 µL per well with Opti‐MEM, according to the manufacturer's protocol. After 48 h, the culture medium was replaced with MEM supplemented with 2% FBS containing SARS‐CoV‐2 at an MOI of 0.01, and the cells were incubated at 37°C for 1 h. Next, the virus‐containing medium was replaced with MEM supplemented with 2% FBS, and the cells were incubated at 37°C for 2 days. On day 2 after infection, culture supernatants were collected to measure viral titer and viral RNA, and the residual cells were fixed with 10% formalin solution (FUJIFILM Wako Pure Chemical Corporation, Osaka, Japan) containing 0.5% crystal violet (FUJIFILM Wako Pure Chemical Corporation) to assess cytopathic effects (CPE). Viral titer was determined by the plaque assay method as described previously [44]. Viral RNA in the collected supernatants was extracted using a QIAamp Viral RNA Mini kit (Qiagen, Hilden, Germany). SARS‐CoV‐2 RdRp RNA levels were quantified by RT‐qPCR using a primer pair specific for the RdRp gene (5′‐CGTCTGCGGTATGTGGAAAG‐3′ and 5′‐TAAGACGGGCTGCACTTACA‐3′) [24] and a QuantiNova SYBR Green RT‐PCR kit (Qiagen) according to the manufacturer's instructions. Relative RdRp RNA levels were calculated relative to the negative control (NC), which was set to 100%. All in vitro experiments handling SARS‐CoV‐2 were conducted under Biosafety Level 3 of Tokushima University.

### 5.8 Statistical Analysis

Data are presented as mean ± SD. Statistical analyses were performed using R software (version 4.3.1). Differences among groups relative to negative control or parental siRNAs were evaluated by a one‐way analysis of variance (ANOVA) followed by Dunnett's multiple‐comparisons test. Sample sizes (*n*) are indicated in the corresponding figure legends. *p* < 0.05 was considered statistically significant. Only statistically significant differences are indicated in the graphs: ^*^ *p* < 0.05, ^**^ *p* < 0.01, and ^***^ *p* < 0.001.

## Conflicts of Interest

The authors declare no conflicts of interest.

## Supporting information



The authors have cited additional references within the Supporting Information [24, 26, 27].
**Supporting File 1**: chem71507‐sup‐0001‐SuppMat.pdf

## Data Availability

The data that support the findings of this study are available in the Supporting Information of this article.
