# Supplementary material for: 4′‐ThioRNA Substitution and Sense‐Strand Segmentation for the Design of Functional Fully Modified siRNAs Targeting SARS‐CoV‐2
Source: Chemistry. 2026 Aug 3;32(40):e71507. doi: 10.1002/chem.71507 (PMC13715996; doi:10.1002/chem.71507)
Supplement: Supplementary file 1 [file CHEM-32-e71507-s001.pdf]

Supporting Information

4'-ThioRNA Substitution and Sense-Strand Segmentation for the Design of Functional Fully Modified siRNAs Targeting SARS-CoV-2.

Yuhei Nogi,[1] Jun Tsukimoto,[1] Noriko Saito-Tarashima,[1]* Takaaki Koma,[2,3] Masako Nomaguchi,[2,3] Noriaki Minakawa[1]*

[1]Graduate School of Pharmaceutical Science, Tokushima University, 1-78-1 Shomachi, Tokushima, Tokushima 770-8505, Japan

[2]Department of Microbiology, Graduate School of Medicine, Tokushima University, 3-18-15 Kuramoto, Tokushima, Tokushima 770-8503, Japan.

[3]Division of Interdisciplinary Researches for Medicine and Photonics, Institute of Post-LED Photonics, Tokushima University, 21 Minamijosanjima, Tokushima, Tokushima 770-8506, Japan.

*Correspondence: noriko.tarashima@tokushima-u.ac.jp (N.S.T); minakawa@tokushima-u.ac.jp (N.M.)

Table of Contents

Page

1. **Figure S1.** CPE-based screening of candidate siRNAs targeting genomic RNA-specific regions of SARS-CoV-2. S3.
2. **Figure S2.** RNAi activities and thermal stabilities of (*S*)-GNA-modified siRNAs. S4.
3. **Figure S3.** CD spectra of the fully 4'-thio-modified sisiRNAs (**sisi75–sisi78**). S5.
4. **Figure S4.** T_m values of the fully 4'-thio-modified sisiRNAs (**sisi75–sisi78**). S6.
5. **Figure S5.** Sense-strand off-target effects of the fully 4'-thio-modified siRNAs (**si62**) and sisiRNAs (**sisi75–sisi78**). S7.
6. **Figure S6.** Nuclease resistance of unmodified **si5** and 4'-thio-modified siRNAs (**si60–si62**, **sisi76**, and **sisi77**). S8.
7. **Figure S7.** Effect of 4'-thio-modified siRNAs and sisiRNAs on SARS-CoV-2 RdRp RNA levels. S9.
8. **Table S1.** Mass analysis of chemically modified oligonucleotides. S10.
9. **Table S2.** Thermal stabilities of siRNAs substituted with a single 4'-thioRNA. S13.

Figure S1. CPE-based screening of candidate siRNAs targeting genomic RNA-specific regions of SARS-CoV-2. Vero E6 cells were pre-transfected with 30.0 nM candidate siRNA **1–9** for 6 h and then infected with SARS-CoV-2 at a multiplicity of infection (MOI) of 0.01. At 2 days post-infection, antiviral activity was evaluated by cytopathic effect (CPE) assay following fixation and crystal violet staining. Representative well images are shown. Retention of crystal violet staining reflects protection against virus-induced cytopathic effects. **si5**, which targets the RdRp (Nsp12) region, consistently exhibited strong antiviral activity and was therefore selected as the model siRNA for subsequent studies. The siRNA sequences used for screening were adopted from previously reported studies as follows: **si1**[24], **si2**[24], **si3**[26], **si4**[24], **si5**[26], **si6**[26] , **si7**[26], **si8**[27], and **si9**[27].

siRNA	Strand	Sequence (5' to 3')	Target genes	CPE assays
**si1**	Sense	CCAAAUGUGCCUUUCAACU	Nsp2	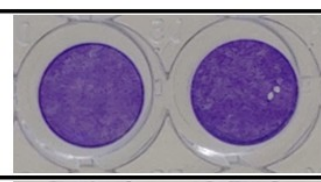
	Antisense	AGUUGAAAGGCACAUUUGGUU		
**si2**	Sense	GUUACAUGCACCAUAUGGA	3CLpro	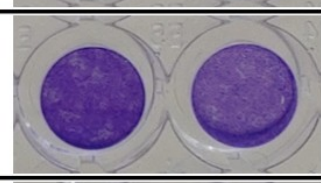
	Antisense	UCCAUAUGGUGCAUGUAACAA		
**si3**	Sense	UGGCUUGAUGACGUAGUUU	3CLpro	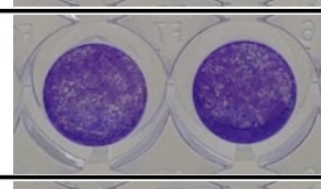
	Antisense	AAACUACGUCAUCAAGCCAUU		
**si4**	Sense	GGUACUUGGUAGUUUAGCU	Nsp9	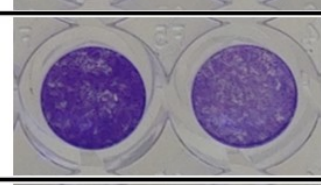
	Antisense	AGCUAAACUACCAAGUACCAU		
**si5**	Sense	CUGUCAAACCCGGUAAUUU	RdRp	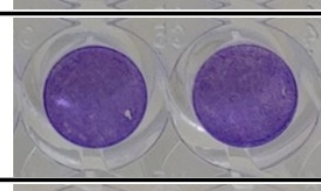
	Antisense	AAAUUACCGGGUUUGACAGUU		
**si6**	Sense	GCGGUUCACUAUAUGUUAA	RdRp	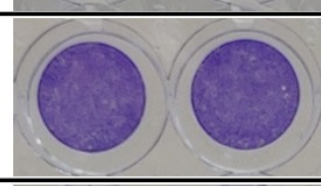
	Antisense	UUAACAUAUAGUGAACCGCUU		
**si7**	Sense	CGCACAUUGCUAACUAAGA	Helicase	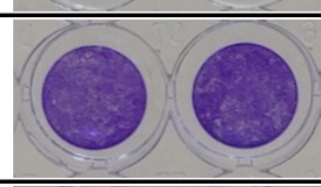
	Antisense	UCUUAGUUAGCAAUGUGCGUU		
**si8**	Sense	CGAAGAAGCUAUAAGACAUGU	Nsp14	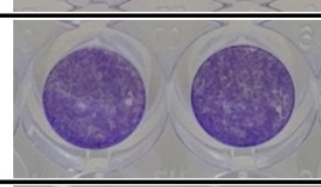
	Antisense	AUGUCUUAUAGCUUCUUCGCG		
**si9**	Sense	CAAUUACCAUUUUUCUAUUAC	Nsp14	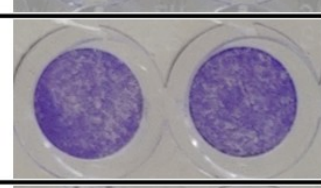
	Antisense	AAUAGAAAAAUGGUAAUUGUU		
Mock (Uninfected)	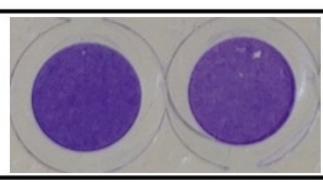		Negative control (NC)	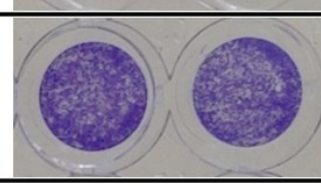

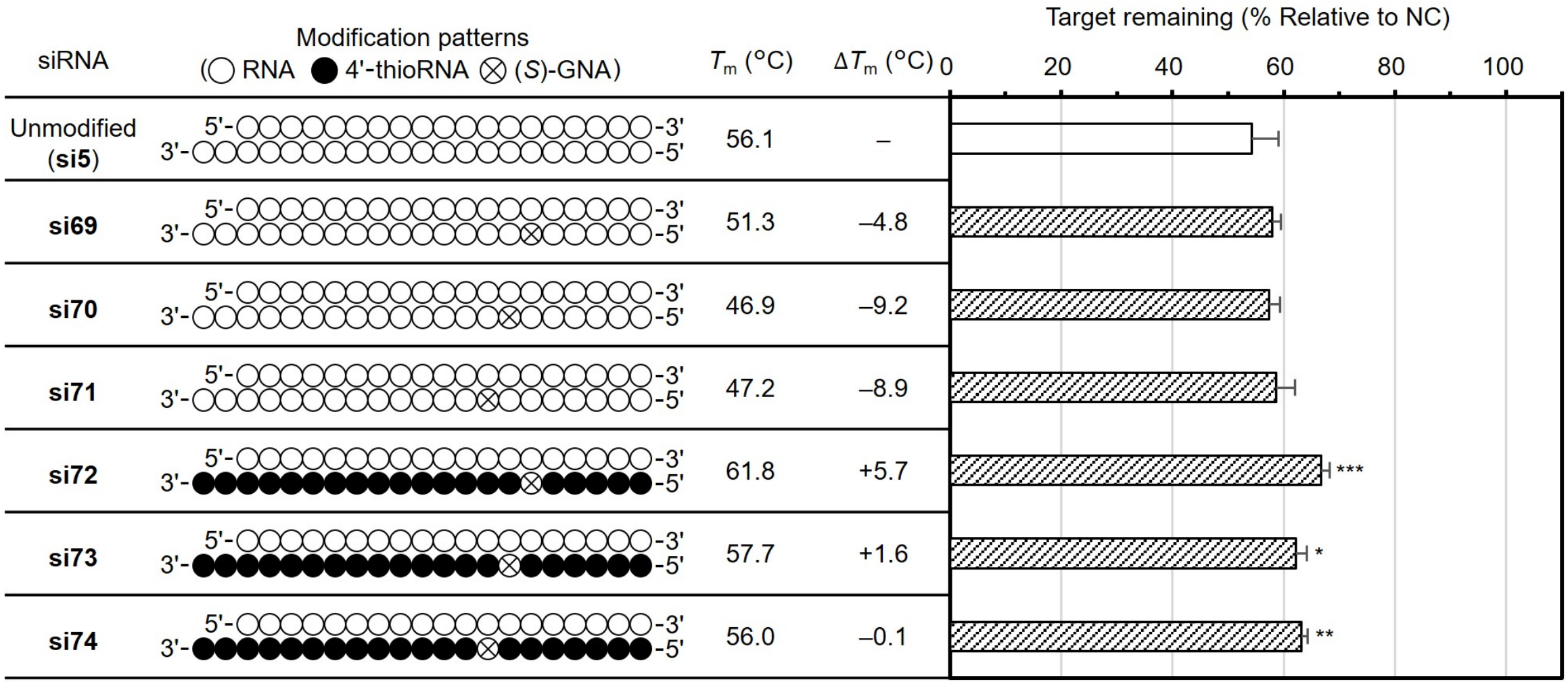

Figure S2. RNAi activities and thermal stabilities of (*S*)-GNA-modified siRNAs. RNAi assays were conducted at 9.00 nM siRNA. Introduction of a single (*S*)-GNA substitution at 6, 7, or 8 from the 5'-end of the antisense strand was tolerated in both unmodified siRNAs (**si69**–**si71**) and siRNAs bearing a 4'-thio-modified antisense strand (**si72**–**si74**). T_m values were determined for siRNA duplexes (1.00 μM) in 0.1×PBS, and ΔT_m values were calculated relative to the unmodified **si5**. Data are shown as mean + SD from three independent replicates (n=3). Statistical significance versus the unmodified **si5** was performed using one-way ANOVA followed by Dunnett's multiple comparisons test; $^{*}P < 0.05$, $^{**}P < 0.01$, $^{***}P < 0.001$.

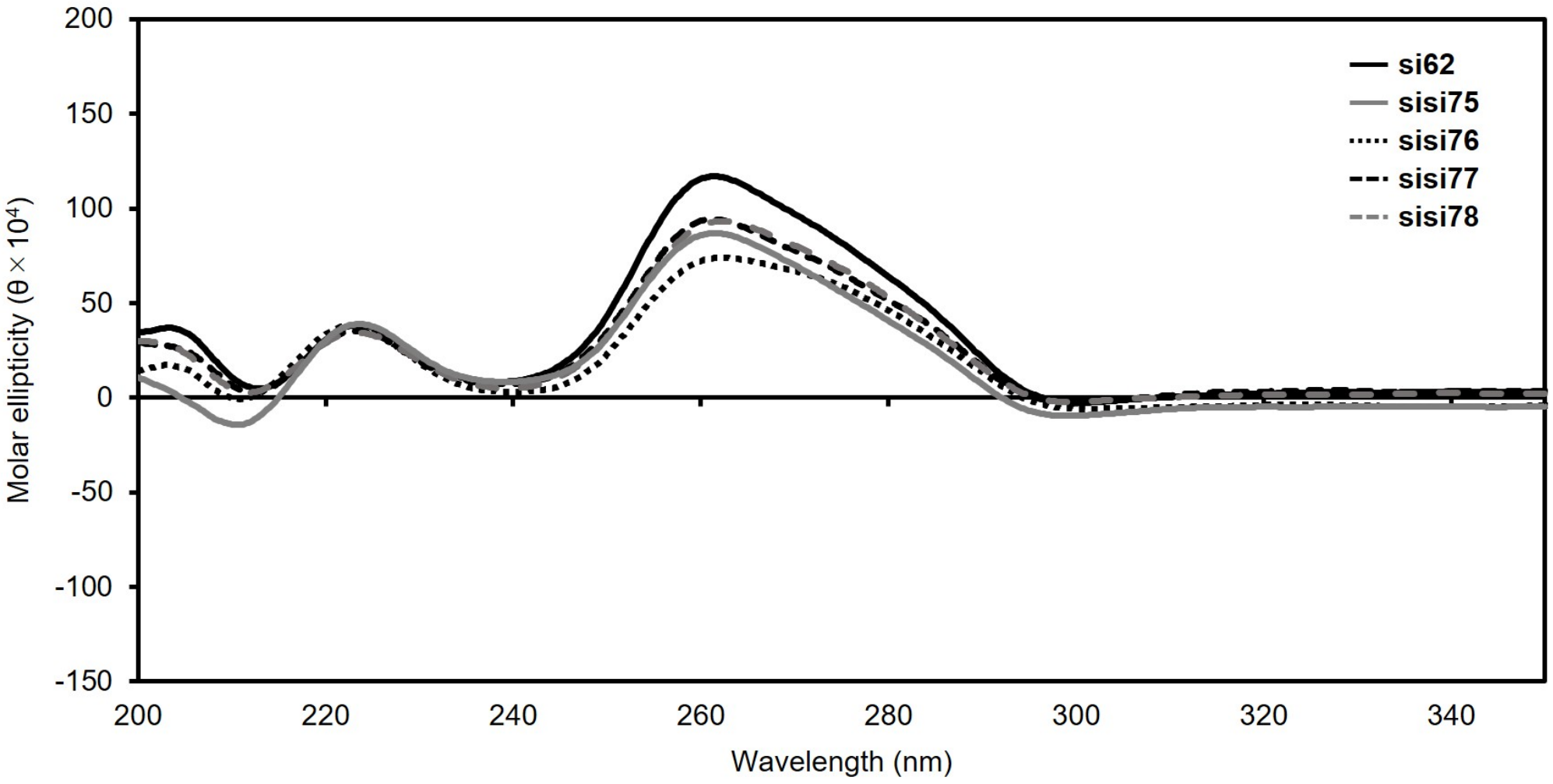

Figure S3. CD spectra of the fully 4'-thio-modified sisiRNAs (**sisi75**–**sisi78**). The spectra were similar to that of the parental **si62**, indicating that introduction of a nick does not substantially alter the global duplex conformation. Solutions contained duplexes with concentration of 2.00 μM in 1×PBS.

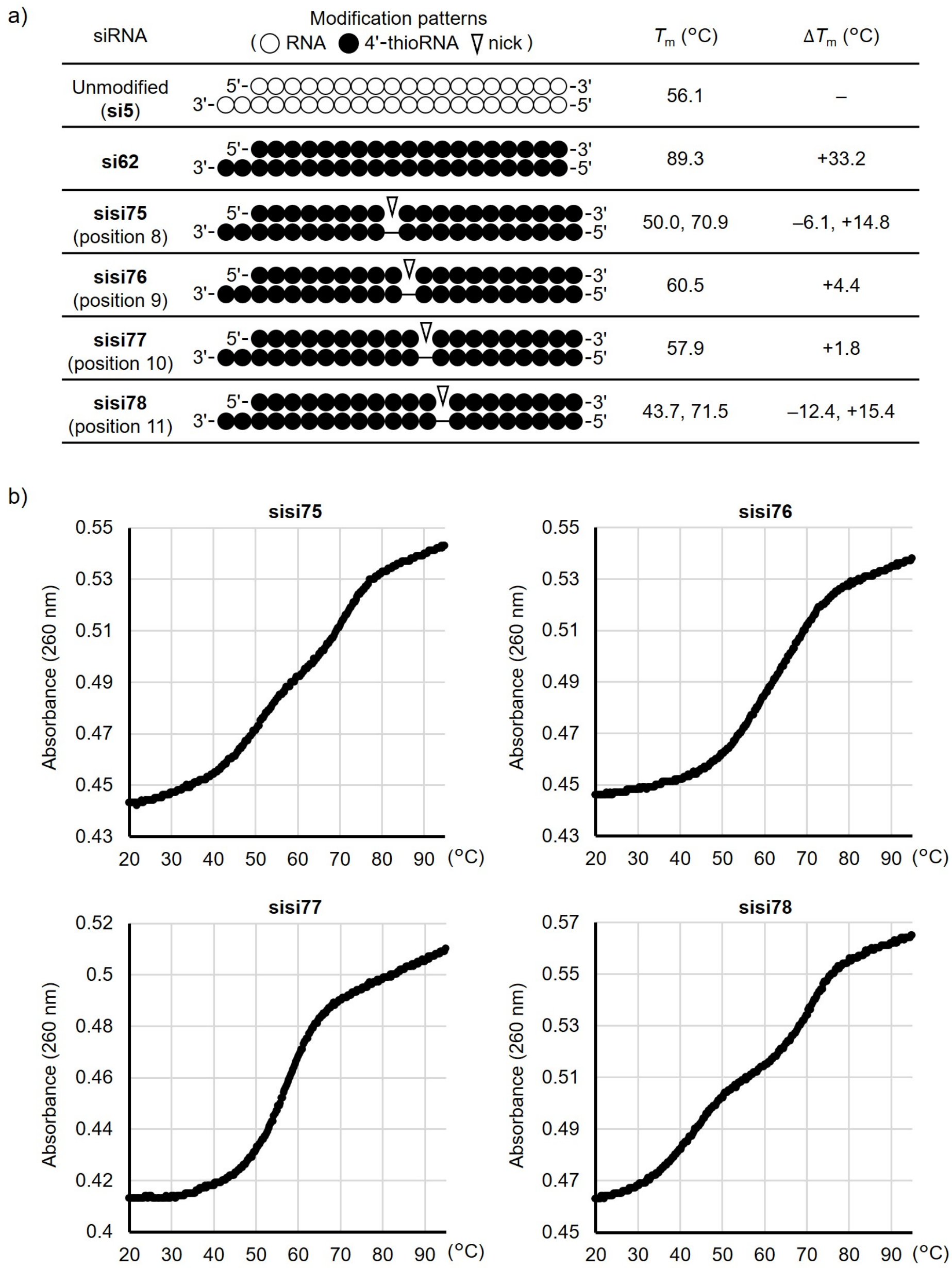

siRNA	Modification patterns (○ RNA ● 4'-thioRNA ∇ nick)	T_m (°C)	ΔT_m (°C)
Unmodified (**si5**)		56.1	–
**si62**		89.3	+33.2
**sisi75** (position 8)		50.0, 70.9	–6.1, +14.8
**sisi76** (position 9)		60.5	+4.4
**sisi77** (position 10)		57.9	+1.8
**sisi78** (position 11)		43.7, 71.5	–12.4, +15.4

Figure S4. T_m values of the fully 4'-thio-modified sisiRNAs (**sisi75–sisi78**). **sisi75–sisi78** exhibited lower T_m values than the parental **si62**. (a) T_m values were determined for siRNA duplexes (1.00 µM) in 0.1×PBS, and ΔT_m values were calculated relative to the unmodified **si5**. (b) UV melting curves of **sisi75–sisi78** monitored at 260 nm.

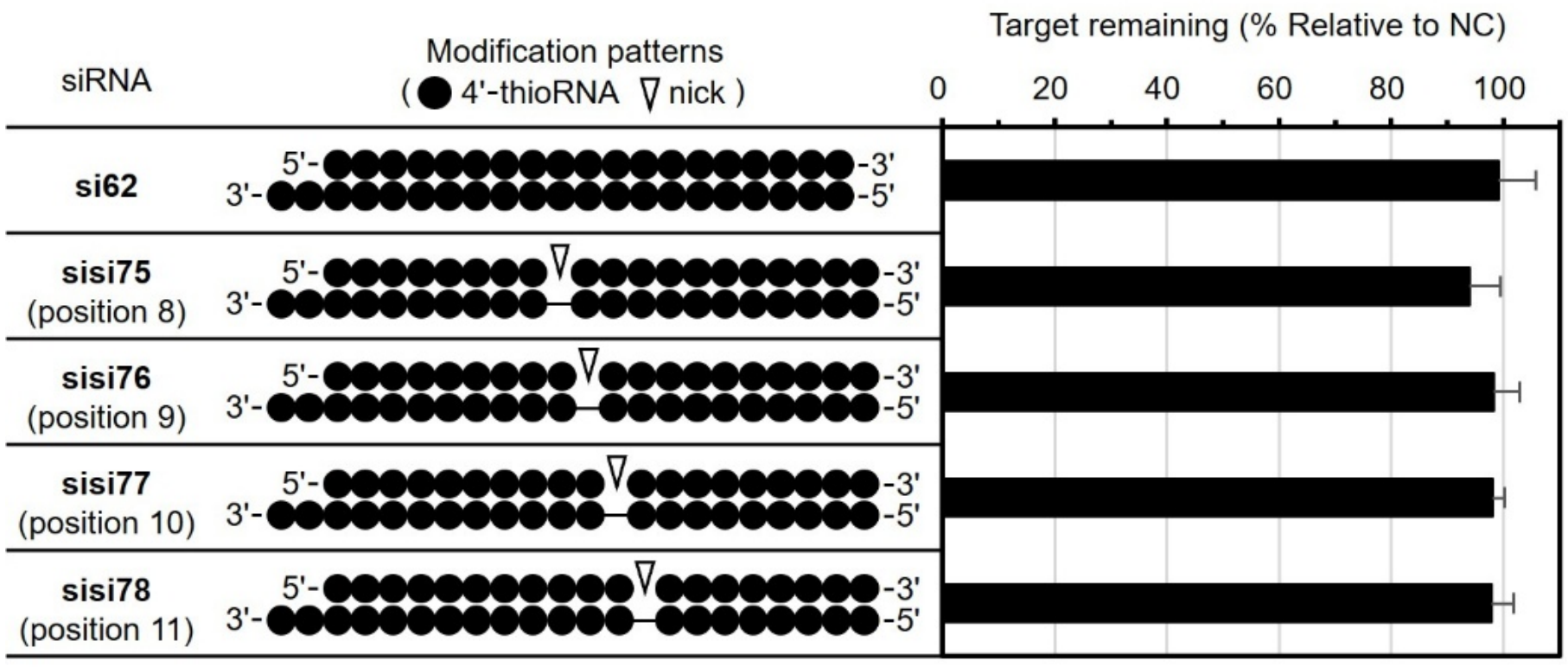

Figure S5. Sense-strand off-target effects of the fully 4'-thio-modified siRNAs (**si62**) and sisiRNAs (**sisi75–sisi78**). No detectable sense-strand-derived silencing activities were observed for **si62** and **sisi75–sisi78**. In this assay, Vero E6 cells were transfected with siRNAs (9.00 nM) and the sense-strand off-target reporter plasmid (100 ng). Luciferase activities were measured at 48 h post-transfection. Data are shown as mean + SD from three independent replicates (n=3).

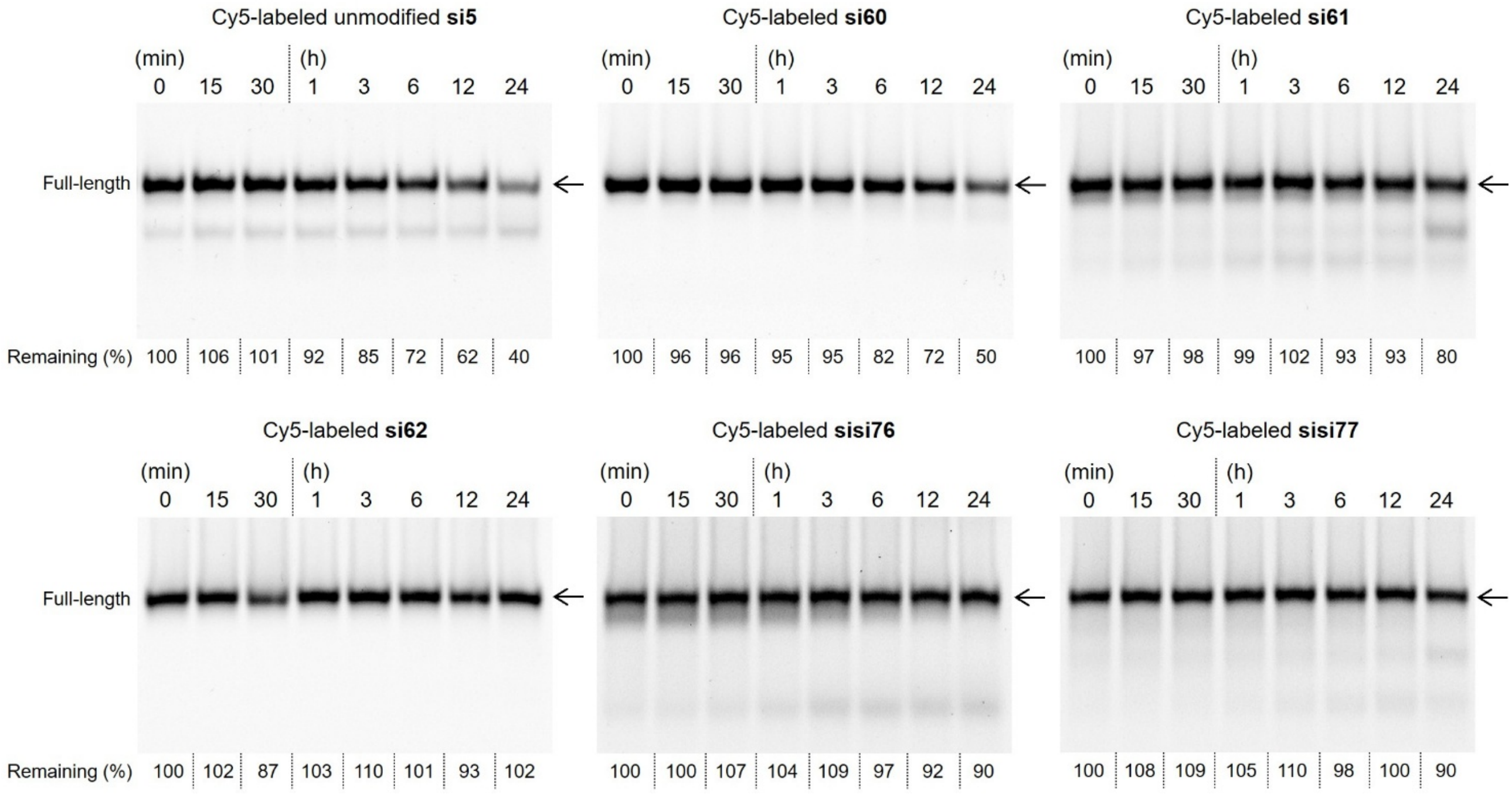

Figure S6. Nuclease resistance of unmodified **si5** and 4'-thio-modified siRNAs (**si60–si62**, **sisi76**, and **sisi77**). Cy5-labeled siRNAs (10.0 pmol) were incubated in 80% FBS at 37 °C for the indicated times, and analyzed by 15% native polyacrylamide gel electrophoresis. Arrows indicate full-length siRNA bands. The percentage of intact siRNA remaining at each time point was quantified from band intensities relative to the 0 min sample.

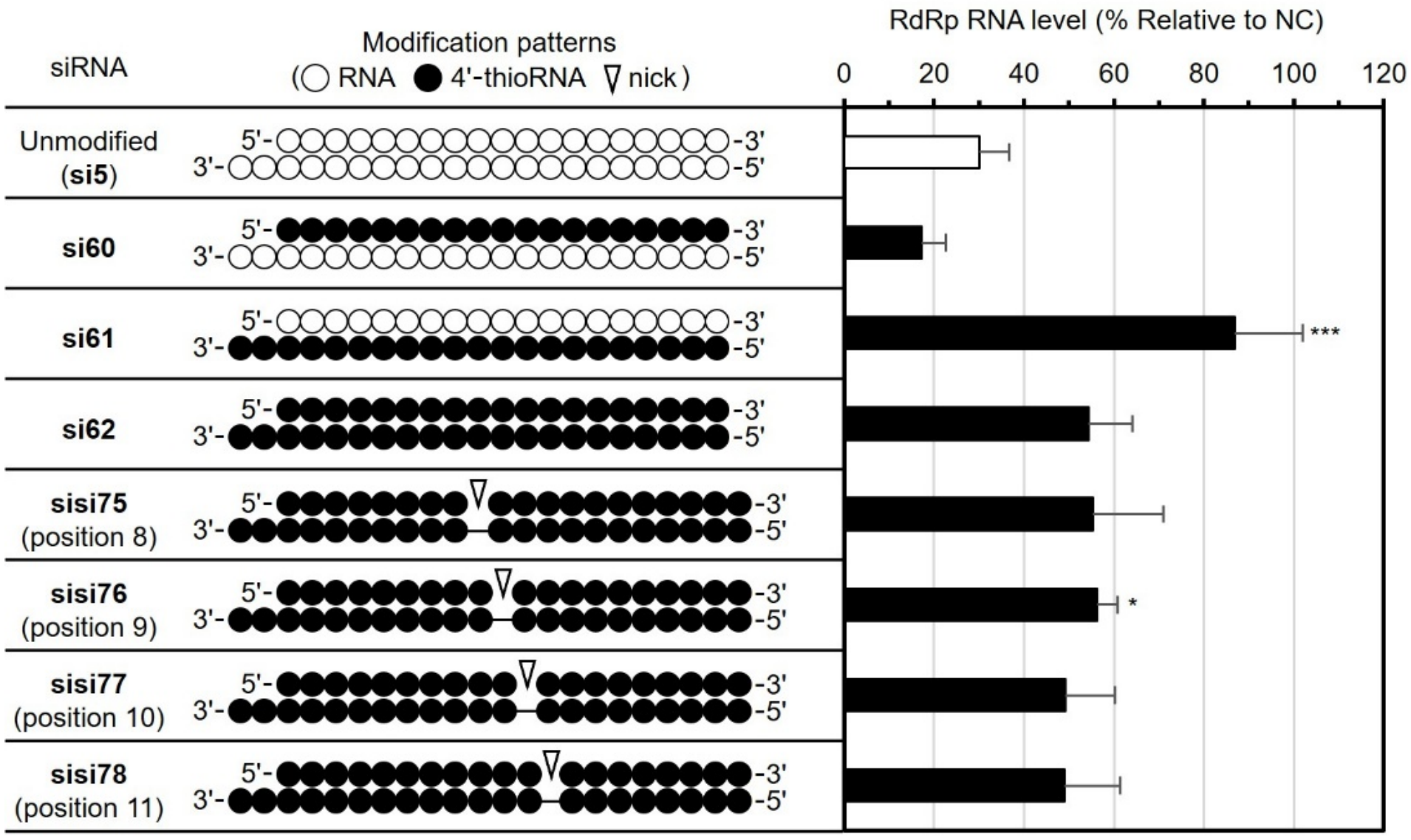

Figure S7. Effect of 4'-thio-modified siRNAs and sisiRNAs on SARS-CoV-2 RdRp RNA levels. Vero E6 cells were pre-transfected with 30.0 nM siRNA for 24 h and then infected with SARS-CoV-2 at an MOI of 0.01. Culture supernatants were collected 2 days after infection, and viral RNA was extracted and quantified by reverse transcription-quantitative polymerase chain reaction (RT-qPCR) targeting the SARS-CoV-2 RdRp gene. Relative RdRp RNA levels were calculated relative to the negative control (NC), which was set to 100%. Data are shown as mean + SD from three independent replicates (n=3). Statistical significance versus unmodified **si5** was assessed using one-way ANOVA followed by Dunnett's multiple comparisons test: **P* < 0.05, ****P* < 0.001.

Table S1. Mass analysis of chemically modified oligonucleotides.

Strand	Sequence (5' to 3')	Formula	Mass data	
			Calculated	Observed
**si10**-Sense	**C**UGUCAAACCCGGUAAUUU	$C_{179}H_{223}N_{67}O_{131}P_{18}S$	5998.6489	5998.7960
**si11**-Sense	C**U**GUCAAACCCGGUAAUUU	$C_{179}H_{223}N_{67}O_{131}P_{18}S$	5998.6489	5998.7951
**si12**-Sense	CU**G**UCAAACCCGGUAAUUU	$C_{179}H_{223}N_{67}O_{131}P_{18}S$	5998.6489	5998.7864
**si13**-Sense	CUG**U**CAAACCCGGUAAUUU	$C_{179}H_{223}N_{67}O_{131}P_{18}S$	5998.6489	5998.8193
**si14**-Sense	CUGU**C**AAACCCGGUAAUUU	$C_{179}H_{223}N_{67}O_{131}P_{18}S$	5998.6489	5998.8807
**si15**-Sense	CUGUC**A**AACCCGGUAAUUU	$C_{179}H_{223}N_{67}O_{131}P_{18}S$	5998.6489	5998.7206
**si16**-Sense	CUGUCA**A**ACCCGGUAAUUU	$C_{179}H_{223}N_{67}O_{131}P_{18}S$	5998.6489	5998.7566
**si17**-Sense	CUGUCAA**A**CCCGGUAAUUU	$C_{179}H_{223}N_{67}O_{131}P_{18}S$	5998.6489	5998.7437
**si18**-Sense	CUGUCAAA**C**CCGGUAAUUU	$C_{179}H_{223}N_{67}O_{131}P_{18}S$	5998.6489	5998.7565
**si19**-Sense	CUGUCAAAC**C**CGGUAAUUU	$C_{179}H_{223}N_{67}O_{131}P_{18}S$	5998.6489	5998.7979
**si20**-Sense	CUGUCAAACC**C**GGUAAUUU	$C_{179}H_{223}N_{67}O_{131}P_{18}S$	5998.6489	5998.5519
**si21**-Sense	CUGUCAAACCC**G**GUAAUUU	$C_{179}H_{223}N_{67}O_{131}P_{18}S$	5998.6489	5998.6804
**si22**-Sense	CUGUCAAACCCG**G**UAAUUU	$C_{179}H_{223}N_{67}O_{131}P_{18}S$	5998.6489	5998.7412
**si23**-Sense	CUGUCAAACCCGG**U**AAUUU	$C_{179}H_{223}N_{67}O_{131}P_{18}S$	5998.6489	5998.7106
**si24**-Sense	CUGUCAAACCCGGU**A**AUUU	$C_{179}H_{223}N_{67}O_{131}P_{18}S$	5998.6489	5998.7774
**si25**-Sense	CUGUCAAACCCGGUA**A**UUU	$C_{179}H_{223}N_{67}O_{131}P_{18}S$	5998.6489	5998.7632
**si26**-Sense	CUGUCAAACCCGGUAA**U**UU	$C_{179}H_{223}N_{67}O_{131}P_{18}S$	5998.6489	5998.7146
**si27**-Sense	CUGUCAAACCCGGUAAU**U**U	$C_{179}H_{223}N_{67}O_{131}P_{18}S$	5998.6489	5998.7090
**si28**-Sense	CUGUCAAACCCGGUAAUU**U**	$C_{179}H_{223}N_{67}O_{131}P_{18}S$	5998.6489	5998.7205
**si29**-Antisense	**A**AAUUACCGGGUUUGACAGUU	$C_{200}H_{246}N_{78}O_{145}P_{20}S$	6714.0691	6714.2368
**si30**-Antisense	A**A**AUUACCGGGUUUGACAGUU	$C_{200}H_{246}N_{78}O_{145}P_{20}S$	6714.0691	6714.1478
**si31**-Antisense	AA**A**UUACCGGGUUUGACAGUU	$C_{200}H_{246}N_{78}O_{145}P_{20}S$	6714.0691	6714.2479
**si32**-Antisense	AAA**U**UACCGGGUUUGACAGUU	$C_{200}H_{246}N_{78}O_{145}P_{20}S$	6714.0691	6714.2432
**si33**-Antisense	AAAU**U**ACCGGGUUUGACAGUU	$C_{200}H_{246}N_{78}O_{145}P_{20}S$	6714.0691	6714.1708
**si34**-Antisense	AAAUU**A**CCGGGUUUGACAGUU	$C_{200}H_{246}N_{78}O_{145}P_{20}S$	6714.0691	6714.1880
**si35**-Antisense	AAAUUA**C**CGGGUUUGACAGUU	$C_{200}H_{246}N_{78}O_{145}P_{20}S$	6714.0691	6714.2029
**si36**-Antisense	AAAUUAC**C**GGGUUUGACAGUU	$C_{200}H_{246}N_{78}O_{145}P_{20}S$	6714.0691	6714.1376
**si37**-Antisense	AAAUUACC**G**GGUUUGACAGUU	$C_{200}H_{246}N_{78}O_{145}P_{20}S$	6714.0691	6714.1041
**si38**-Antisense	AAAUUACCG**G**GUUUGACAGUU	$C_{200}H_{246}N_{78}O_{145}P_{20}S$	6714.0691	6714.1179
**si39**-Antisense	AAAUUACCGG**G**UUUGACAGUU	$C_{200}H_{246}N_{78}O_{145}P_{20}S$	6714.0691	6714.1227
**si40**-Antisense	AAAUUACCGGG**U**UUGACAGUU	$C_{200}H_{246}N_{78}O_{145}P_{20}S$	6714.0691	6714.1639
**si41**-Antisense	AAAUUACCGGGU**U**UGACAGUU	$C_{200}H_{246}N_{78}O_{145}P_{20}S$	6714.0691	6714.1810
**si42**-Antisense	AAAUUACCGGGUU**U**GACAGUU	$C_{200}H_{246}N_{78}O_{145}P_{20}S$	6714.0691	6714.2591

**si43**-Antisense	AAAUUACCGGGUUU**G**ACAGUU	$C_{200}H_{246}N_{78}O_{145}P_{20}S$	6714.0691	6714.1472
**si44**-Antisense	AAAUUACCGGGUUUG**A**CAGUU	$C_{200}H_{246}N_{78}O_{145}P_{20}S$	6714.0691	6714.2487
**si45**-Antisense	AAAUUACCGGGUUUGA**C**AGUU	$C_{200}H_{246}N_{78}O_{145}P_{20}S$	6714.0691	6714.1314
**si46**-Antisense	AAAUUACCGGGUUUGAC**A**GUU	$C_{200}H_{246}N_{78}O_{145}P_{20}S$	6714.0691	6714.1491
**si47**-Antisense	AAAUUACCGGGUUUGACA**G**UU	$C_{200}H_{246}N_{78}O_{145}P_{20}S$	6714.0691	6714.1064
**si48**-Antisense	AAAUUACCGGGUUUGACAG**U**U	$C_{200}H_{246}N_{78}O_{145}P_{20}S$	6714.0691	6714.2577
**si49**-Antisense	AAAUUACCGGGUUUGACAGU**U**	$C_{200}H_{246}N_{78}O_{145}P_{20}S$	6714.0691	6714.2492
**si50**-Sense	**CUGUC**AAACCCGGUAAUUU	$C_{179}H_{223}N_{67}O_{127}P_{18}S_5$	6062.9113	6062.6790
**si51**-Sense	CUGUC**AAACCC**GGUAAUUU	$C_{179}H_{223}N_{67}O_{126}P_{18}S_6$	6078.9769	6078.6474
**si52**-Sense	CUGUCAAACCC**GGUAAUUU**	$C_{179}H_{223}N_{67}O_{124}P_{18}S_8$	6111.1081	6111.5159
**si53**-Antisense	**AAAUUACC**GGGUUUGACAGUU	$C_{200}H_{246}N_{78}O_{138}P_{20}S_8$	6826.5283	6826.8671
**si54**-Antisense	AAAUUACC**GGGUUU**GACAGUU	$C_{200}H_{246}N_{78}O_{140}P_{20}S_6$	6794.3971	6794.1253
**si55**-Antisense	AAAUUACCGGGUUU**GACAGUU**	$C_{200}H_{246}N_{78}O_{139}P_{20}S_7$	6810.4627	6810.1037
**si56**-Sense	**C**U**G**U**C**A**A**A**C**C**C**G**G**U**A**A**U**U**U**	$C_{179}H_{223}N_{67}O_{122}P_{18}S_{10}$	6143.2393	6143.6457
**si57**-Sense	C**U**G**U**C**A**A**A**C**C**C**G**G**U**A**A**U**U**U	$C_{179}H_{223}N_{67}O_{123}P_{18}S_9$	6127.1737	6127.6109
**si58**-Antisense	**A**A**A**U**U**A**C**C**G**G**G**U**U**U**G**A**C**A**G**U**U**	$C_{200}H_{246}N_{78}O_{135}P_{20}S_{11}$	6874.7251	6874.9895
**si59**-Antisense	A**A**A**U**U**A**C**C**G**G**G**U**U**U**G**A**C**A**G**U**U	$C_{200}H_{246}N_{78}O_{136}P_{20}S_{10}$	6858.6595	6858.9902
**si60**-Sense	**CUGUCAAACCCGGUAAUUU**	$C_{179}H_{223}N_{67}O_{113}P_{18}S_{19}$	6287.8297	6287.3557
**si61**-Antisense	**AAAUUACCGGGUUUGACAGUU**	$C_{200}H_{246}N_{78}O_{125}P_{20}S_{21}$	7035.3811	7034.8374
**si63**-Sense	cugucaaacccgguaauuu	$C_{198}H_{261}N_{67}O_{132}P_{18}$	6249.0883	6249.1919
**si64**-Antisense	aaauuaccggguuugacaguu	$C_{221}H_{288}N_{78}O_{146}P_{20}$	6992.5616	6992.6713
**si66**-Antisense	**AAAUU***A***CCGGGUUUGACAGUU**	$C_{198}H_{244}N_{78}O_{124}P_{20}S_{20}$	6961.2794	6961.8497
**si67**-Antisense	**AAAUUA***C***CGGGUUUGACAGUU**	$C_{198}H_{244}N_{78}O_{124}P_{20}S_{20}$	6961.2794	6961.8994
**si68**-Antisense	**AAAUUAC***C***GGGUUUGACAGUU**	$C_{198}H_{244}N_{78}O_{124}P_{20}S_{20}$	6961.2794	6961.8909
**si69**-Antisense	AAAUU*A*CCGGGUUUGACAGUU	$C_{198}H_{244}N_{78}O_{144}P_{20}$	6639.9674	6640.1108
**si70**-Antisense	AAAUUA*C*CGGGUUUGACAGUU	$C_{198}H_{244}N_{78}O_{144}P_{20}$	6639.9674	6640.1528
**si71**-Antisense	AAAUUAC*C*GGGUUUGACAGUU	$C_{198}H_{244}N_{78}O_{144}P_{20}$	6639.9674	6640.2212
**sisi75**-Sense	**CUGUCAAA**	$C_{76}H_{95}N_{30}O_{45}P_7S_8$	2622.0778	2622.1852
**sisi75**-Sense	**CCCGGUAAUUU**	$C_{103}H_{129}N_{37}O_{66}P_{10}S_{11}$	3603.7873	3603.0546
**sisi76**-Sense	**CUGUCAAAC**	$C_{85}H_{107}N_{33}O_{51}P_8S_9$	2943.3247	2942.9734
**sisi76**-Sense	**CCGGUAAUUU**	$C_{94}H_{117}N_{34}O_{60}P_9S_{10}$	3282.5404	3281.8256
**sisi77**-Sense	**CUGUCAAACC**	$C_{94}H_{119}N_{36}O_{57}P_9S_{10}$	3264.5715	3263.9023
**sisi77**-Sense	**CGGUAAUUU**	$C_{85}H_{105}N_{31}O_{54}P_8S_9$	2961.2936	2960.7165
**sisi78**-Sense	**CUGUCAAACCC**	$C_{103}H_{131}N_{39}O_{63}P_{10}S_{11}$	3585.8184	3586.4955
**sisi78**-Sense	**GGUAAUUU**	$C_{76}H_{93}N_{28}O_{48}P_7S_8$	2640.0467	2639.4683
**si5**-Cy5-Antisense	AAAUUACCGGGUUUGACAGUU-Cy5	$C_{231}H_{284}N_{80}O_{150}P_{21}$	7231.6216	7230.7879

**si61**-Cy5-Antisense	**<u>AAAUUACCGGGUUUGACAGUU</u>**-Cy5	$C_{231}H_{284}N_{80}O_{129}P_{21}S_{21}$	7568.9992	7569.4576

Uppercase, uppercase bold underlined letters, lower case and italicized uppercase represent RNA, 4'-thioRNA, 2'-OMe and (*S*)-GNA modifications, respectively.

Table S2. Thermal stabilities of siRNAs substituted with a single 4'-thioRNA.

siRNA	Sequence[a]	T_m (°C)[b]	ΔT_m (°C)[c]
Unmodified (**si5**)	5'-CUGUCAAACCCGGUAAUUU-3'<br>3'-UUGACAGUUUGGGCCAUUAAA-5'	56.1	–
**si10**	5'-**C**UGUCAAACCCGGUAAUUU-3'<br>3'-UUGACAGUUUGGGCCAUUAAA-5'	56.4	+0.3
**si11**	5'-C**U**GUCAAACCCGGUAAUUU-3'<br>3'-UUGACAGUUUGGGCCAUUAAA-5'	57.3	+1.2
**si12**	5'-CU**G**UCAAACCCGGUAAUUU-3'<br>3'-UUGACAGUUUGGGCCAUUAAA-5'	58.0	+1.9
**si13**	5'-CUG**U**CAAACCCGGUAAUUU-3'<br>3'-UUGACAGUUUGGGCCAUUAAA-5'	57.3	+1.2
**si14**	5'-CUGU**C**AAACCCGGUAAUUU-3'<br>3'-UUGACAGUUUGGGCCAUUAAA-5'	56.6	+0.5
**si15**	5'-CUGUC**A**AACCCGGUAAUUU-3'<br>3'-UUGACAGUUUGGGCCAUUAAA-5'	57.2	+1.1
**si16**	5'-CUGUCA**A**ACCCGGUAAUUU-3'<br>3'-UUGACAGUUUGGGCCAUUAAA-5'	56.3	+0.2
**si17**	5'-CUGUCAA**A**CCCGGUAAUUU-3'<br>3'-UUGACAGUUUGGGCCAUUAAA-5'	56.2	+0.1
**si18**	5'-CUGUCAAA**C**CCGGUAAUUU-3'<br>3'-UUGACAGUUUGGGCCAUUAAA-5'	56.1	0.0
**si19**	5'-CUGUCAAAC**C**CGGUAAUUU-3'<br>3'-UUGACAGUUUGGGCCAUUAAA-5'	56.9	+0.8
**si20**	5'-CUGUCAAACC**C**GGUAAUUU-3'<br>3'-UUGACAGUUUGGGCCAUUAAA-5'	57.4	+1.3
**si21**	5'-CUGUCAAACCC**G**GUAAUUU-3'<br>3'-UUGACAGUUUGGGCCAUUAAA-5'	57.6	+1.5
**si22**	5'-CUGUCAAACCCG**G**UAAUUU-3'<br>3'-UUGACAGUUUGGGCCAUUAAA-5'	58.6	+2.5
**si23**	5'-CUGUCAAACCCGG**U**AAUUU-3'<br>3'-UUGACAGUUUGGGCCAUUAAA-5'	57.0	+0.9
**si24**	5'-CUGUCAAACCCGGU**A**AUUU-3'<br>3'-UUGACAGUUUGGGCCAUUAAA-5'	56.4	+0.3
**si25**	5'-CUGUCAAACCCGGUA**A**UUU-3'<br>3'-UUGACAGUUUGGGCCAUUAAA-5'	56.7	+0.6

**si26**	5'-CUGUCAAACCCGGUAA**U**UU-3'<br>3'-UUGACAGUUUGGGCCAUUAAA-5'	56.5	+0.4
**si27**	5'-CUGUCAAACCCGGUAAU**U**U-3'<br>3'-UUGACAGUUUGGGCCAUUAAA-5'	56.4	+0.3
**si28**	5'-CUGUCAAACCCGGUAAUU**U**-3'<br>3'-UUGACAGUUUGGGCCAUUAAA-5'	56.9	+0.8
**si29**	5'-CUGUCAAACCCGGUAAUUU-3'<br>3'-UUGACAGUUUGGGCCAUUAA**A**-5'	56.8	+0.7
**si30**	5'-CUGUCAAACCCGGUAAUUU-3'<br>3'-UUGACAGUUUGGGCCAUUA**A**A-5'	56.8	+0.7
**si31**	5'-CUGUCAAACCCGGUAAUUU-3'<br>3'-UUGACAGUUUGGGCCAUU**A**AA-5'	57.3	+1.2
**si32**	5'-CUGUCAAACCCGGUAAUUU-3'<br>3'-UUGACAGUUUGGGCCAU**U**AAA-5'	57.0	+0.9
**si33**	5'-CUGUCAAACCCGGUAAUUU-3'<br>3'-UUGACAGUUUGGGCCA**U**UAAA-5'	57.4	+1.3
**si34**	5'-CUGUCAAACCCGGUAAUUU-3'<br>3'-UUGACAGUUUGGGCC**A**UUAAA-5'	57.1	+1.0
**si35**	5'-CUGUCAAACCCGGUAAUUU-3'<br>3'-UUGACAGUUUGGGC**C**AUUAAA-5'	56.4	+0.3
**si36**	5'-CUGUCAAACCCGGUAAUUU-3'<br>3'-UUGACAGUUUGGG**C**CAUUAAA-5'	57.4	+1.3
**si37**	5'-CUGUCAAACCCGGUAAUUU-3'<br>3'-UUGACAGUUUGG**G**CCAUUAAA-5'	57.4	+1.3
**si38**	5'-CUGUCAAACCCGGUAAUUU-3'<br>3'-UUGACAGUUUG**G**GCCAUUAAA-5'	58.9	+2.8
**si39**	5'-CUGUCAAACCCGGUAAUUU-3'<br>3'-UUGACAGUUU**G**GGCCAUUAAA-5'	57.9	+1.8
**si40**	5'-CUGUCAAACCCGGUAAUUU-3'<br>3'-UUGACAGUU**U**GGGCCAUUAAA-5'	57.4	+1.3
**si41**	5'-CUGUCAAACCCGGUAAUUU-3'<br>3'-UUGACAGU**U**UGGGCCAUUAAA-5'	56.9	+0.8
**si42**	5'-CUGUCAAACCCGGUAAUUU-3'<br>3'-UUGACAG**U**UUGGGCCAUUAAA-5'	57.5	+1.4
**si43**	5'-CUGUCAAACCCGGUAAUUU-3'<br>3'-UUGACA**G**UUUGGGCCAUUAAA-5'	57.1	+1.0

**si44**	5'-CUGUCAAACCCGGUAAUUU-3'<br>3'-UUGAC**<u>A</u>**GUUUGGGCCAUUAAA-5'	56.9	+0.8
**si45**	5'-CUGUCAAACCCGGUAAUUU-3'<br>3'-UUGA**<u>C</u>**AGUUUGGGCCAUUAAA-5'	56.3	+0.2
**si46**	5'-CUGUCAAACCCGGUAAUUU-3'<br>3'-UUG**<u>A</u>**CAGUUUGGGCCAUUAAA-5'	56.8	+0.7
**si47**	5'-CUGUCAAACCCGGUAAUUU-3'<br>3'-UU**<u>G</u>**ACAGUUUGGGCCAUUAAA-5'	57.0	+0.9
**si48**	5'-CUGUCAAACCCGGUAAUUU-3'<br>3'-U**<u>U</u>**GACAGUUUGGGCCAUUAAA-5'	57.3	+1.2
**si49**	5'-CUGUCAAACCCGGUAAUUU-3'<br>3'-**<u>U</u>**UGACAGUUUGGGCCAUUAAA-5'	57.6	+1.5

[a]Uppercase and uppercase bold underlined letters represent RNA and 4'-thioRNA, respectively. [b]T_m values were determined for siRNA duplexes (1.00 μM) in 0.1×PBS. [c]ΔT_m values were calculated relative to the unmodified **si5**.
